# Efficacy of acupuncture, Chinese herbal medicine, combined acupuncture-herbal therapy, and western medication for adult chronic insomnia: a network meta-analysis

**DOI:** 10.3389/fmed.2026.1961539

**Published:** 2026-09-30

**Authors:** Ning Yang, Yunxi Li, Jinying Zhao, Rulin Wang, Sixian Wang, Zhijie Li, Zhihao Dong, Hailin Jiang, Fuchun Wang

**Affiliations:** 1 Acupuncture and Massage College, Changchun University of Chinese Medicine, Changchun, Jilin, China; 2 Shenzhen Traditional Chinese Medicine Hospital, Shenzhen, Guangdong, China

**Keywords:** acupuncture and pharmacotherapy, chronic insomnia, network meta-analysis, sleep quality, total efficacy rate

## Abstract

**Background:**

Chronic insomnia is a prevalent sleep disorder that significantly impairs quality of life and reduces patient confidence in treatment. Acupuncture, Chinese herbal medicine, and their combination have shown promise in the management of this condition. However, comparative research on these Chinese medicine modalities themselves and vs. Western medication remains scarce.

**Methods:**

PubMed, EMBASE, Cochrane Library, Web of Science, CNKI, Wanfang, VIP, and the Chinese Biomedical Literature Database were comprehensively searched from database inception to June 1, 2026. Two reviewers independently screened studies, extracted data, and assessed the risk of bias in the eligible studies. Network meta-analysis was conducted using STATA 16 and R 4.5.3.

**Results:**

Fifty-nine randomized controlled trials (RCTs) involving 4 interventions were included. According to the surface under the cumulative ranking curve (SUCRA) analysis, for total efficacy (TE), acupuncture plus herbal medicine ranked the highest (92.0%), followed by acupuncture (74.7%), herbal medicine (33.1%), and Western medicine (0.2%). For Pittsburgh Sleep Quality Index (PSQI), acupuncture ranked first (82.0%), followed by acupuncture plus herbal medicine (76.8%), herbal medicine (36.4%), and Western medicine (4.9%). For adverse events, where a higher SUCRA indicates fewer events, acupuncture ranked first (78.8%), followed by herbal medicine (76.0%), acupuncture plus herbal medicine (45.2%), and Western medicine (<0.1%).

**Conclusion:**

Combination therapy (acupuncture plus herbal medicine) showed the highest probability of being the most effective intervention for total efficacy based on SUCRA analysis, and significantly outperformed Western medication and herbal medicine alone. However, no significant difference was observed between combination therapy and acupuncture alone. Acupuncture alone ranked first for improving sleep quality (PSQI) and minimizing adverse events. Given the methodological limitations of the included studies, high-quality, rigorously designed RCTs are warranted to validate these findings.

**Systematic Review Registration:**

https://www.crd.york.ac.uk/prospero/display_record.php?ID=CRD420261413406, Identifier CRD420261413406.

## 1 Background

Chronic insomnia is a highly prevalent sleep disorder characterized by persistent difficulty initiating or maintaining sleep, occurring at least three nights per week for a minimum of three months, with accompanying daytime functional impairment (1). Studies from the field of epidemiology show that between 10%–15% of adults are diagnosed with long-term insomnia, a condition that is more commonly found among females and senior citizens (2). Beyond its direct impact on sleep architecture, chronic insomnia profoundly impairs health-related quality of life, reduces patients’ confidence in treatment, and imposes a substantial psychological and socioeconomic burden (3). In addition, a growing body of research confirms that long-term sleeplessness stands alone as a risk factor for mental health conditions, especially for disorders like depression and anxiety, along with heart and metabolic issues (4). The pathophysiology of chronic insomnia is thought to involve a state of conditioned hyperarousal, characterized by an imbalance of sleep-promoting and wake-promoting neurotransmitters—particularly reduced gamma-aminobutyric acid (GABA) activity and excessive glutamatergic signaling—together with dysregulation of the sleep-wake circuitry governed by the hypothalamus and the circadian system, and hyperactivity of the hypothalamic-pituitary-adrenal (HPA) axis (5–7). Glutamatergic signaling through NMDA receptors is a key driver of arousal, and its dysregulation has been implicated in sleep-wake disturbances and related neuropsychiatric conditions (8). Insomnia is also highly prevalent among older adults and frequently co-occurs with chronic pain and other somatic conditions that further disrupt sleep (9). Moreover, experimental evidence indicates that sleep deprivation itself induces neuroinflammation and gut microbiota dysregulation, which may in turn aggravate sleep and cognitive disturbances (10).

Recent medical protocols advocate for Cognitive Behavioral Therapy for Insomnia (CBT-I) as the preferred initial intervention; nonetheless, its extensive application is hindered by limited availability, financial constraints, and fluctuating levels of patient compliance (11). The use of medication for the treatment of sleep disorders such as benzodiazepines, melatoninergic enhancers, and compounds that restrict the activity of two orexin receptors remains prevalent in medical practice (12). While these drugs provide temporary alleviation of symptoms, their effectiveness over time is often limited due to negative side effects including lingering daytime drowsiness, diminished cognitive function, increased tolerance, addiction potential, and repercussions associated with discontinuation of use (13). These constraints highlight the pressing requirement for secure, bearable, and potent supplementary or alternative treatments.

Acupuncture and Chinese herbal medicine, two core modalities of traditional Chinese medicine, have demonstrated considerable promise in the management of chronic insomnia (14). Based on the concepts of comprehensive treatment and pattern identification, these treatments strive to reestablish the equilibrium between yin and yang and to facilitate the coordination of organ activities, thereby enhancing sleep quality (15). Recent empirical findings and in-depth research indicate the potential of acupuncture to adjust levels of neurotransmitters like gamma-aminobutyric acid (GABA) and serotonin, amidst the broader effects of medicinal herbs from China that are associated with inducing relaxation and enhancing sleep via various pathways (16, 17).

Despite growing research interest, the comparative effectiveness of acupuncture, Chinese herbal medicine, and their combination vs. Western pharmacotherapy remains insufficiently characterized (18). Existing pairwise meta-analyses have typically focused on single comparisons, leaving a gap in the holistic evaluation of all available Chinese medicine modalities. Therefore, a comprehensive synthesis using network meta-analysis is warranted to simultaneously compare multiple interventions and rank their relative efficacy based on key outcome measures (19). This study aimed to evaluate the comparative efficacy of acupuncture, Chinese herbal medicine, their combination, and Western medicine in treating chronic insomnia through a network meta-analysis of randomized controlled trials.

## 2 Data and methods

### 2.1 Methods

This network meta-analysis was conducted in accordance with the Preferred Reporting Items for Systematic Reviews and Meta-Analyses (PRISMA), including the PRISMA extension for network meta-analyses (20). The study protocol was prospectively registered with the International Prospective Register of Systematic Reviews (PROSPERO) (CRD420261413406).

### 2.2 Search strategy

PubMed, Embase, Cochrane Library, Web of Science, CNKI, Wanfang Data, VIP, and the Chinese Biomedical Literature Database (CBM) were comprehensively searched from database inception to June 1, 2026 to collect related publications written in English and Chinese. Medical subject headings (MeSH) and free-text terms were combined to design search terms. MeSH terms encompassed chronic insomnia, acupuncture therapy, Chinese herbal medicine, hypnotics and sedatives. Additionally, reference lists of relevant studies and gray literature sources were manually screened to identify additional eligible studies. The full search strategy is provided in Appendix 1.

### 2.3 Eligibility criteria

Studies were eligible for inclusion if they met the following criteria: (1) Participants: Adults with chronic insomnia as the primary complaint, diagnosed according to the International Classification of Sleep Disorders, Third Edition (ICSD-3) (21) or other recognized diagnostic criteria explicitly reported in the original trials; studies in which insomnia was attributable to a specific comorbid condition (e.g., perimenopausal or diabetic insomnia) were eligible only when insomnia was the primary treatment target and outcomes were reported independently. (2) Interventions: Included studies compared at least two of the following four interventions: acupuncture therapy (including manual acupuncture with filiform needles, electroacupuncture, warm needle acupuncture, auricular acupuncture, and other acupuncture techniques that involve needle insertion; pure moxibustion or thermal methods without needling were excluded because they are typically indicated only for specific traditional Chinese medicine patterns in chronic insomnia, and their mechanism differs substantially from needle-based stimulation), Chinese herbal medicine (including decoctions, granules, capsules, etc.), combined acupuncture-herbal therapy, and Western medication (including benzodiazepines, non-benzodiazepine hypnotics, sedating antidepressants, etc.). Trials that did not include at least two of these four interventions (e.g., those comparing an intervention solely against placebo or sham control without another eligible active comparator) were excluded. (3) Study design: Randomized controlled trials (RCTs) only. (4) Outcomes: Primary outcomes encompassed sleep quality as measured by the Pittsburgh Sleep Quality Index (PSQI) total score and total efficacy (TE). TE was assessed based on predefined criteria, such as PSQI score reduction thresholds or guideline-recommended standards. The secondary outcome was adverse events. Specifically, TE was assessed according to the standardized four-level grading criteria recommended by the Guiding Principles for Clinical Research of New Chinese Medicines and related guideline-recommended standards—clinically cured, markedly effective, effective, and ineffective—graded by the percentage reduction in PSQI score (typically ≥75%, 50%–74%, 25%–49%, and <25%, respectively) or, in older trials, by the percentage improvement in nighttime sleep duration. A participant reaching any of the first three grades was classified as a responder. For RR computation, the event was defined as non-response (ineffective); consequently, an RR < 1 indicates a lower probability of non-response, i.e., higher efficacy.

Studies were excluded if they met any of the following criteria: (1) non-clinical studies (e.g., animal or *in vitro* experiments), case reports, study protocols, reviews, letters, editorials, or conference abstracts; (2) studies with incomplete or seriously flawed data; (3) duplicate publications; (4) full text unavailable; or (5) overlapping or duplicate participants.

### 2.4 Data extraction

All retrieved records were imported into EndNote. Two reviewers (Yang and Zhao) independently screened titles and abstracts against the predefined eligibility criteria, and then reviewed the full texts of potentially eligible reports. Any discrepancies were resolved through discussion or consultation with a third reviewer (Li).

Data were independently extracted by two reviewers (Yang and Li) using a standardized electronic extraction form. Extracted variables included author name, year of publication, study location, intervention and control details, treatment duration, baseline characteristics of participants, and outcome measures.

### 2.5 Quality assessment

The methodological quality of included studies was independently assessed by two reviewers (Yang and Li) using the Cochrane Risk of Bias 2.0 (ROB 2.0) tool (22). This tool assesses five domains: the randomization process, deviations from intended interventions, missing outcome data, measurement of the outcome, and selection of the reported result. Each domain was rated as low risk of bias (RoB), some concerns, or high RoB. The overall risk of bias for a study was considered low if all domains were rated low risk or if only one domain raised some concerns. A study was considered high risk if any domain was rated high or if four or more domains raised some concerns. Studies that did not meet either criterion were classified as having some concerns. Disagreements were resolved through discussion or consultation with a third reviewer (Jiang).

### 2.6 Statistical analysis

All statistical analyses were performed using STATA version 16 and R version 4.5.3. Mean differences (MDs) with 95% credible intervals (CrIs) were calculated for continuous outcomes (Pittsburgh Sleep Quality Index), and risk ratios (RRs) with 95% CrIs were calculated for total efficacy, and odds ratios (ORs) with 95% CrIs were calculated for adverse events. Statistical heterogeneity was assessed with the Cochrane I^2^ statistic. I^2^ values > 50% indicated substantial heterogeneity. Random-effects models were applied when heterogeneity was present; otherwise, fixed-effects models were used. Publication bias was assessed visually using comparison-adjusted funnel plots.

For the network meta-analysis, the assumptions of transitivity, homogeneity, and consistency must be met. Transitivity was assessed qualitatively by comparing baseline characteristics across studies. Homogeneity was tested using the mtc.anohe function in the GeMTC package, with I^2^ < 50% indicating acceptable heterogeneity. Consistency between direct and indirect evidence was examined using node-splitting (mtc.nodesplit function in GeMTC), where *P* > 0.05 indicated no significant inconsistency. Convergence was evaluated using convergence diagnostics, with a Gelman-Rubin statistic of 1 indicating ideal convergence. The NMA was fitted using Bayesian hierarchical consistency models implemented in the GeMTC package, with a normal likelihood and identity link for the continuous outcome (PSQI) and a binomial likelihood with log (RR) or logit (OR) link for binary outcomes (TE and AEs, respectively). Vague priors were assigned following the GeMTC defaults: relative treatment effects were given a normal prior centered at 0 with a large, data-derived standard deviation {d∼N[0, (15 x om.scale)^2^], where om.scale is estimated from the data, e.g., sd = 96 for PSQI}, and, in random-effects models, the between-study standard deviation was given a uniform prior U(0, om.scale). Four Markov chains were run, each with 10,000 adaptation (burn-in) iterations followed by 50,000 sampling iterations, with a thinning interval of 1, yielding 200,000 retained samples for posterior inference. Node-splitting models were fitted with random effects using 5,000 adaptation iterations and 100,000 sampling iterations (thinning=1). Convergence was verified using the Gelman-Rubin potential scale reduction factor (PSRF) and inspection of trace and density plots. The full node-splitting results for each outcome are provided in Appendix 4.

Network plots were generated, with nodes representing interventions and edges representing direct comparisons. Node size corresponded to the number of trials per intervention, and edge thickness corresponded to the number of comparisons. Interventions were ranked using the surface under the cumulative ranking curve (SUCRA), representing the probability of each intervention being the most effective. All network graphs were generated using RStudio version 4.5.3.

Meta-regression was performed to explore potential sources of heterogeneity for total efficacy and PSQI. Covariates included sample size, publication year, treatment duration, and mean patient age. Meta-regression was not conducted for adverse events due to the limited number of studies (*n* = 10) reporting this outcome.

## 3 Results

### 3.1 Literature search and study selection

A total of 2,748 articles were initially retrieved from the databases. After removing 1,445 duplicates, the titles and abstracts of 1,303 records were screened, and 1,145 were excluded. The full texts of the remaining 158 articles were assessed for eligibility, of which 99 were excluded for the following reasons: lack of eligible outcome indicators (*n* = 58), study design not meeting the inclusion criteria (*n* = 38), and duplicate publications (*n* = 3). Ultimately, 59 studies were included in the analysis. The study selection process is illustrated in Figure 1.

**Figure 1:**
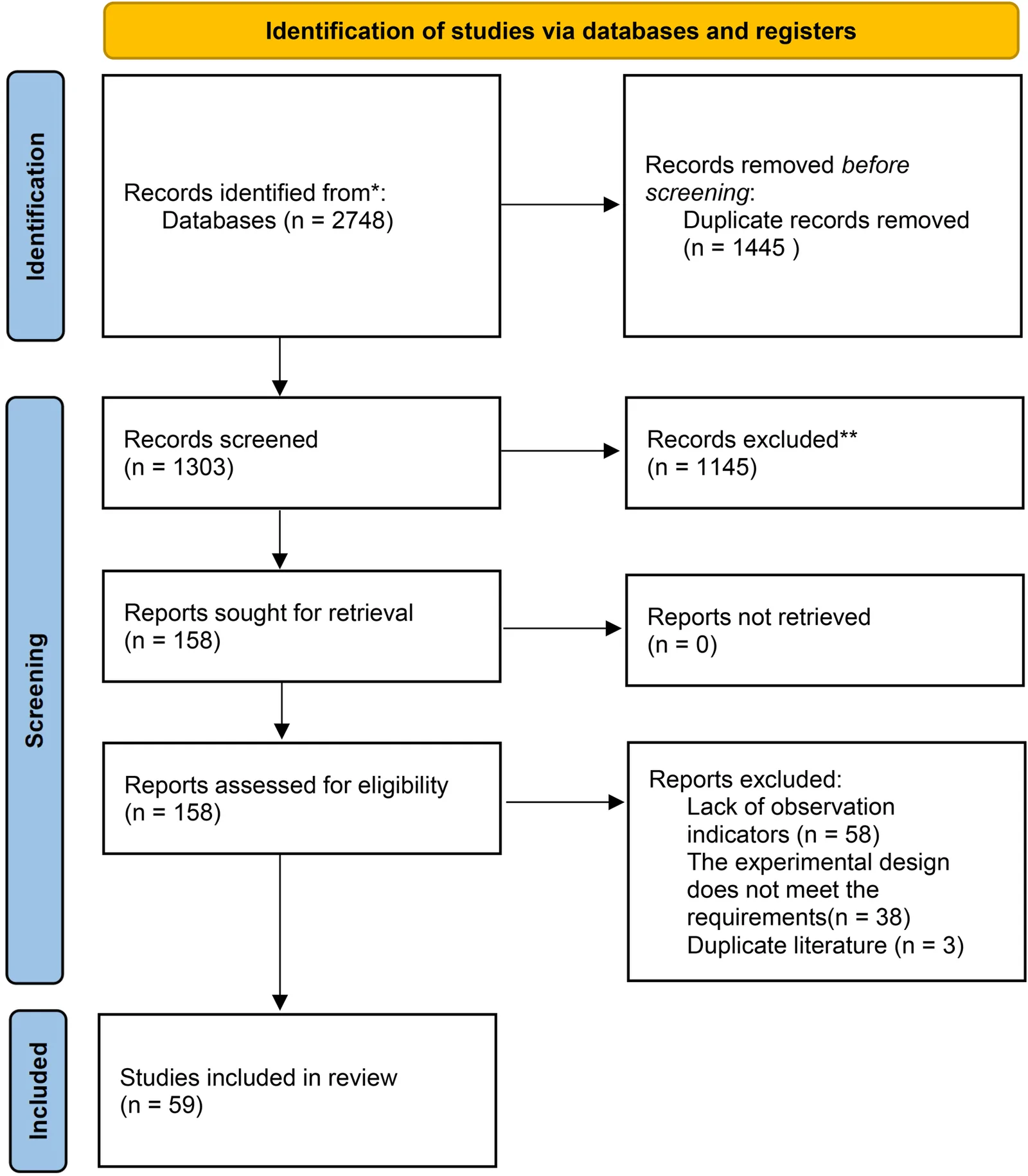
The process of literature identification and selection.

### 3.2 Characteristics and quality assessment of included studies

The 59 included studies were RCTs (23–81), comprising 5,070 participants (2,554 in the intervention groups and 2,516 in the control groups). Participants were adults aged 18 years and older, with mean ages ranging from approximately 35 to 70 years across studies. All trials were conducted in China and published between 2003 and 2025, and all were two-arm comparisons among the four target interventions. Across these studies, Western medication was evaluated as a treatment arm in 44 RCTs, acupuncture in 30 RCTs, herbal medicine in 20 RCTs, and acupuncture plus herbal medicine in 24 RCTs. Detailed study characteristics are summarized in Supplementary Table 2 and Appendix 2.

The quality of the included studies was appraised using the Cochrane Risk of Bias 2.0 (RoB 2.0) tool across its five domains—the randomization process, deviations from intended interventions, missing outcome data, measurement of the outcome, and selection of the reported result—and the rationale underlying the rating of each domain is described below. For the randomization process, 41 of the 59 RCTs explicitly reported the method used to generate the random allocation sequence—most commonly a random number table, with a small number of trials using computer-generated random numbers, drawing of lots, or allocation according to the order of clinic visits—whereas the remaining 18 studies (23, 25, 28, 30, 32–35, 50, 51, 53, 54, 64, 68–71, 75) merely stated that participants were “randomly allocated” without specifying the method; in addition, none of the included trials explicitly reported whether the allocation sequence was concealed (e.g., central randomization or sequentially numbered, opaque, sealed envelopes). Because it could not be verified that group assignments were unforeseeable at the time of enrollment, the randomization process was rated as some concerns for all studies, which may introduce a risk of selection bias. For deviations from intended interventions, because reliable blinding of participants and practitioners is inherently infeasible in acupuncture trials, none of the included RCTs employed blinding, and this domain was rated accordingly. For missing outcome data, studies with low attrition that was balanced between groups, or in which missing data were appropriately handled, were rated as low risk, whereas studies with non-negligible or unexplained dropout were rated as having some concerns. For measurement of the outcome, because the primary outcomes (TE and PSQI) are subjective, patient-reported measures susceptible to detection bias in the absence of blinding, this domain was judged to materially increase the risk of bias. For selection of the reported result, because none of the included trials provided a prospectively registered protocol or statistical analysis plan, selective outcome reporting could not be ruled out, and this domain was generally rated as some concerns (Figure 2).

**Figure 2:**
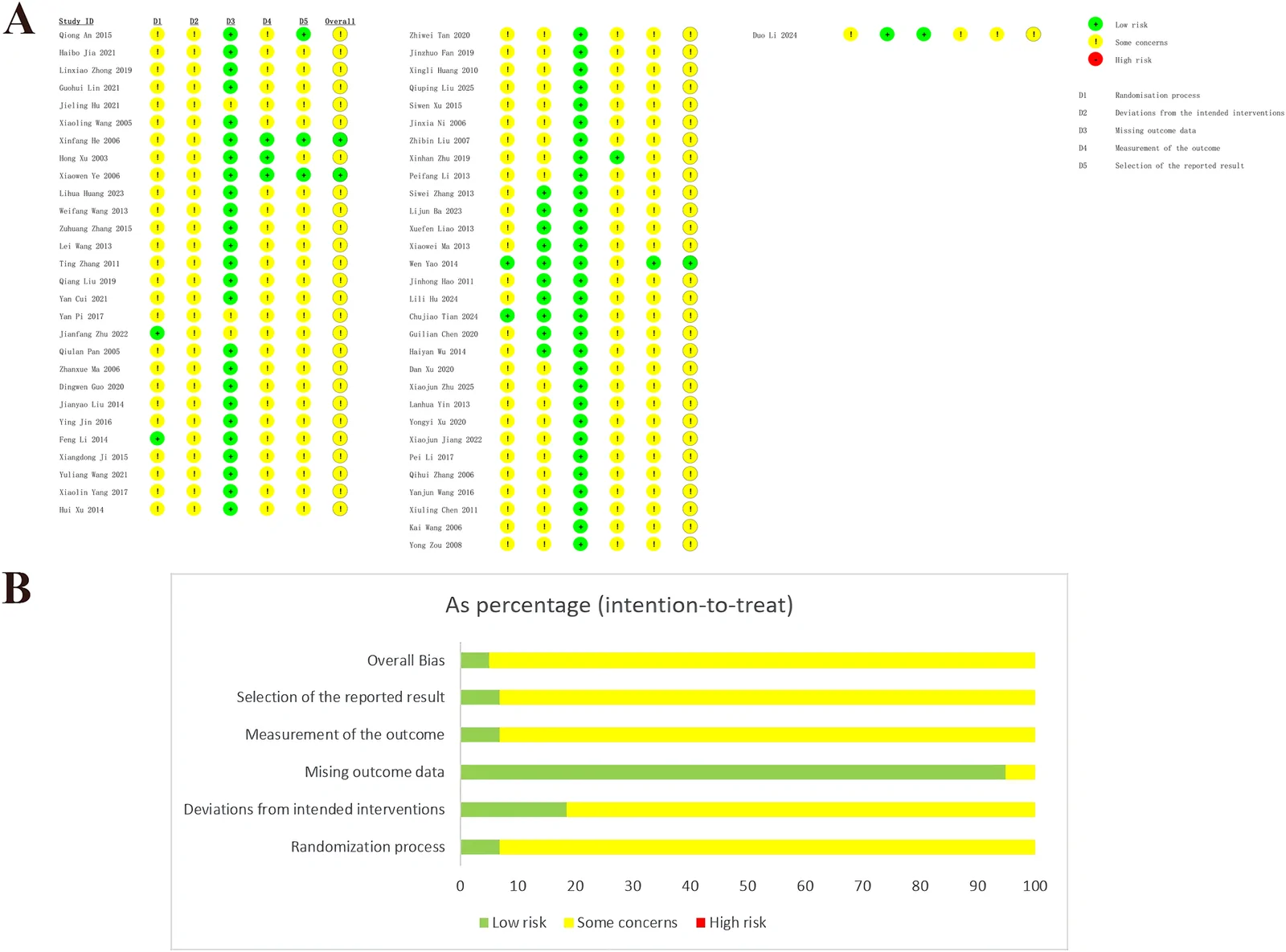
Assessment of the risk of bias in the included studies using the RoB 2.0 tool: **(A)** distribution across specific domains and the overall risk of bias; **(B)** Bar graph showing the weighted distribution of risk of bias ratings.

### 3.3 Network diagram

Within the network diagram, each node symbolizes a separate intervention, with the size of the nodes correlating to the frequency of research on that intervention. Connecting lines denote one-to-one comparisons, and the extent of their thickness is indicative of the comparative study count (Figure 3). The potential scale reduction factor (PSRF) for all outcomes was 1.00, indicating satisfactory model convergence.

**Figure 3:**
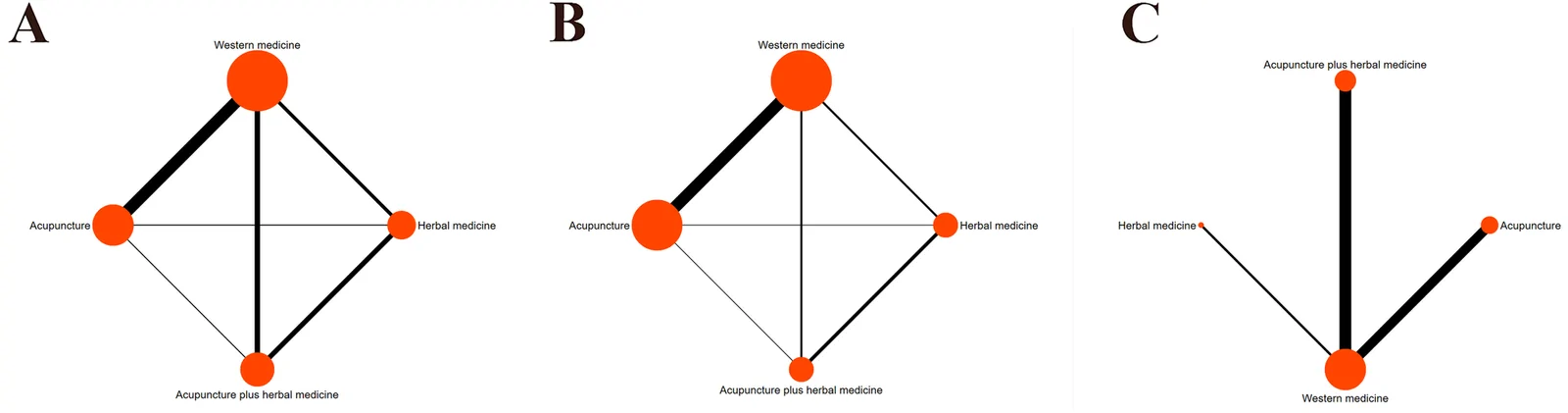
Network diagrams for treatment comparisons: **(A)** TE; **(B)** PSQI; **(C)** AEs.

### 3.4 Total efficacy (TE)

A total of 56 studies involving 4,856 patients reported TE. With moderate heterogeneity (I^2^ = 35%), a fixed-effects model was applied. Meta-regression analysis was performed to explore potential moderators of effect size variation (see Section 3.7). Pooled results indicated that acupuncture plus herbal medicine was significantly more effective than Western medicine [RR = 0.83; 95% CrI: (0.79, 0.87)] and herbal medicine [RR = 0.88; 95% CrI: (0.84, 0.92)], whereas no significant difference was found between acupuncture plus herbal medicine and acupuncture alone [RR = 0.98; 95% CrI: (0.93, 1.04)]. Acupuncture was significantly more effective than herbal medicine [RR = 1.12; 95% CrI: (1.06, 1.18)], and Western medicine was significantly less effective than acupuncture [RR = 0.84; 95% CrI: (0.81, 0.87)]. Herbal medicine was significantly more effective than Western medicine [RR = 0.94; 95% CrI: (0.90, 0.99)] and significantly less effective than acupuncture plus herbal medicine [RR = 0.88; 95% CrI: (0.84, 0.92)]. (Table 1) It should be noted that the event used for RR computation was non-response (ineffective); therefore, an RR < 1 indicates a lower probability of non-response and hence higher efficacy for the favored intervention. Node-splitting analysis revealed statistically significant inconsistency between direct and indirect evidence for two comparisons (herbal medicine vs. Western medicine, *P* = 0.022; acupuncture plus herbal medicine vs. Western medicine, *P* = 0.008) and a borderline result for acupuncture plus herbal medicine vs. herbal medicine (*P* = 0.050); no significant inconsistency was detected for the remaining comparisons (Appendix 4). The TE results for these comparisons should therefore be interpreted with caution.

**Table 1:** League table for TE.

Treatment				
**Western medicine**	**Western medicine**			
**Acupuncture**	**0.84** (**0.81, 0.87)**	**Acupuncture**		
**Herbal medicine**	**0.94** (**0.9, 0.99)**	**1.12 (1.06, 1.18)**	**Herbal medicine**	
**Acupuncture plus herbal medicine**	**0.83** (**0.79, 0.87)**	0.98 (0.93, 1.04)	**0.88 (0.84, 0.92)**	**Acupuncture plus herbal medicine**

RR (95% CrI) of column vs. row. RR < 1 favors row (higher efficacy in the row intervention); RR > 1 favors column (higher efficacy in the column intervention). Bold=significant (95% CrI excludes 1). For total efficacy, higher response rate indicates better outcome. The event for RR computation was non-response (ineffective); thus, RR < 1 indicates a lower non-response rate (i.e., higher efficacy).

The SUCRA ranking of TE was as follows: acupuncture plus herbal medicine (92.0%) > acupuncture (74.7%) > herbal medicine (33.1%) > Western medicine (0.2%). (Figure 4A and Appendix 3).

**Figure 4:**
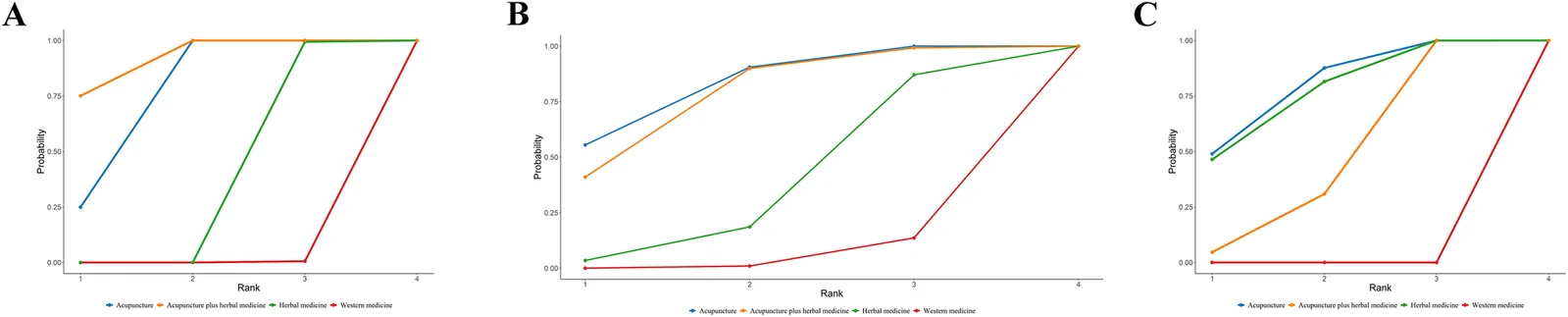
Ranking curves showing cumulative probabilities: **(A)** TE; **(B)** PSQI; **(C)** AEs.

### 3.5 Pittsburgh sleep quality index (PSQI)

Thirty studies involving 2,675 participants reported PSQI. Substantial heterogeneity was observed (I^2^ = 96%), and a random-effects model was applied. The sources of this heterogeneity were explored through meta-regression analysis (see Section 3.7). Pooled results indicated that acupuncture significantly reduced PSQI scores compared with Western medicine [MD = 2.45; 95% CrI: (1.29, 3.62)], as did acupuncture plus herbal medicine [MD = 2.26; 95% CrI: (0.24, 4.29)]. No significant differences were observed among acupuncture, herbal medicine, and acupuncture plus herbal medicine. (Table 2).

**Table 2:** League table for PSQI.

Treatment				
**Western medicine**	**Western medicine**			
**Acupuncture**	**2.45** (**1.29, 3.62)**	**Acupuncture**		
**Herbal medicine**	1.13 (−0.89, 3.14)	−1.33 (−3.51, 0.84)	**Herbal medicine**	
**Acupuncture plus herbal medicine**	**2.26** (**0.24, 4.29)**	−0.19 (−2.37, 1.98)	1.14 (−0.74, 3.03)	**Acupuncture plus herbal medicine**

MD (95% CrI) of column minus row. A positive value indicates the row intervention has a lower PSQI score (better sleep quality) than the column; a negative value indicates the column intervention has a lower PSQI score than the row. Bold=significant (95% CrI excludes 0). For PSQI, lower scores indicate better sleep quality.

The SUCRA ranking for PSQI was as follows: acupuncture (82.0%) > acupuncture plus herbal medicine (76.8%) > herbal medicine (36.4%) > Western medicine (4.9%). (Figure 4B and Appendix 3).

### 3.6 Adverse events (AEs)

Ten studies involving 788 participants reported adverse events. Given minimal heterogeneity (I^2^ = 16%), a fixed-effects model was applied. Pooled results indicated that Western medicine was associated with significantly higher odds of adverse events compared with acupuncture [OR=8.82; 95% CrI: (3.04, 39.32)], herbal medicine [OR=8.43; 95% CrI: (2.40, 59.37)], and acupuncture plus herbal medicine [OR=4.45; 95% CrI: (2.59, 8.60)]. No significant differences were found among acupuncture, herbal medicine, and acupuncture plus herbal medicine. (Table 3) Adverse events were defined as any untoward medical occurrence reported during the treatment period, regardless of its suspected causal relationship to the intervention, and were extracted as the number of participants experiencing at least one event per arm. Commonly reported events included daytime drowsiness, dizziness, and gastrointestinal discomfort for pharmacological treatments, and mild subcutaneous bleeding, bruising, or transient needling pain for acupuncture. It should be acknowledged that adverse-event ascertainment was not standardized across trials: most studies recorded adverse events through spontaneous patient reports and investigator observation during follow-up visits rather than through prospective, protocol-defined monitoring with validated instruments. For AEs, node-splitting was not applicable because no comparison in the network was informed by both direct and indirect evidence (Appendix 4).

**Table 3:** League table for AEs.

Treatment				
**Western medicine**	**Western medicine**			
**Acupuncture**	**8.82** (**3.04, 39.32)**	**Acupuncture**		
**Herbal medicine**	**8.43** (**2.4, 59.37)**	0.96 (0.14, 8.65)	**Herbal medicine**	
**Acupuncture plus herbal medicine**	**4.45** (**2.59, 8.6)**	0.51 (0.1, 1.77)	0.53 (0.07, 2.19)	**Acupuncture plus herbal medicine**

OR (95% CrI) of column vs. row. OR > 1 indicates higher odds of adverse events in the column intervention than in the row (worse safety for column, better for row); OR < 1 indicates lower odds in the column than in the row (better safety for column, worse for row). Bold=significant (95% CrI excludes 1).

The SUCRA ranking for AEs, where higher values indicate a lower risk of adverse events (better safety profile), was as follows: acupuncture (78.8%) > herbal medicine (76.0%) > acupuncture plus herbal medicine (45.2%) > Western medicine (<0.1%). (Figure 4C and Appendix 3).

### 3.7 Meta-regression analysis

To discern potential sources of heterogeneity, a meta-regression analysis was conducted, focusing on total effectiveness and the Pittsburgh Sleep Quality Index (PSQI), considering variables such as sample size, publication year, treatment duration, and patient age. Among these, sample size was identified as the principal influential factor in the PSQI context (beta=2.129; 95% CrI: 0.429, 3.859), significantly reducing the I^2^ value from 96% to 0.5%. The study indicates that the noted substantial variability in PSQI outcomes is mainly attributed to differences in statistical power among different studies. Notably, larger sample size studies tend to demonstrate greater improvements in PSQI scores. For overall efficacy, the publication year emerged as the only significant moderator (beta=1.018; 95% CrI: 0.350, 1.666), implying newer studies are more likely to report higher effect sizes. The treatment duration and patient age did not significantly alter the outcomes in comparison. Meta-regression analysis on adverse events was not feasible due to the limited number of studies supplying this data (*n* = 10), which precluded precise analysis.

### 3.8 Publication bias

Referencing Figure 5, the funnel plots for total efficacy and adverse events appeared broadly symmetrical, suggesting no obvious publication bias for these outcomes. However, the funnel plot for PSQI showed some asymmetry, which—together with the meta-regression finding that sample size was the primary source of heterogeneity—suggests the possible presence of small-study effects; the PSQI results should therefore be interpreted with caution. An Egger regression test was additionally performed for PSQI, yielding *P* = 0.078, which indicates no statistically significant small-study effect at the 0.05 level, although a borderline trend toward asymmetry cannot be excluded; the PSQI results are therefore interpreted together with the funnel plot and meta-regression findings above.

**Figure 5:**
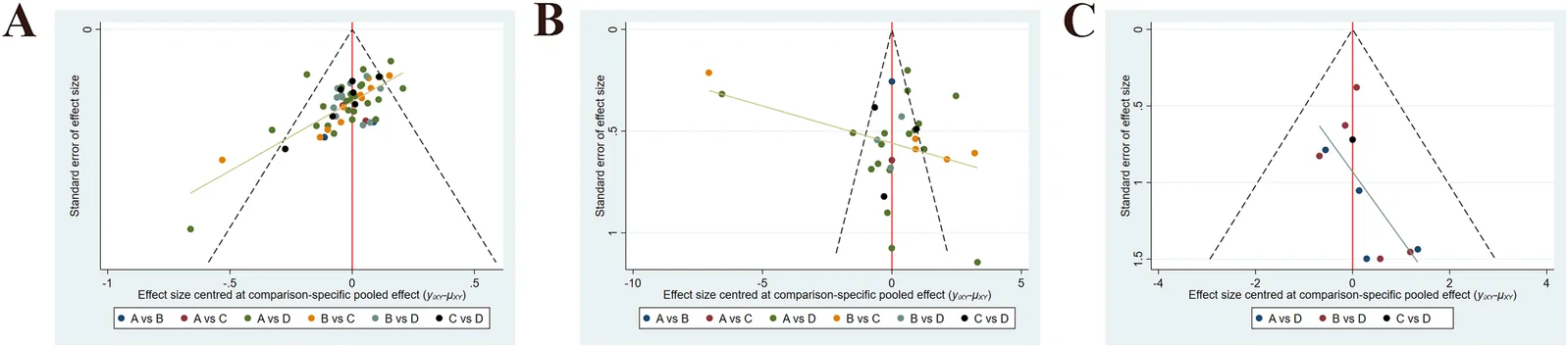
Funnel plots. **(A)** TE; **(B)** PSQI; **(C)** AEs.

## 4 Discussion

This systematic review and network meta-analysis comprehensively evaluated the comparative efficacy and safety of four distinct treatment strategies—acupuncture, Chinese herbal medicine, combined acupuncture-herbal therapy, and Western medication—for adult chronic insomnia. A total of 59 RCTs involving 5,070 participants were included. The analysis focused on total efficacy, PSQI scores, and adverse events to explore the clinical effectiveness of these interventions.

The rationale for grouping the interventions into four broad categories, rather than subdividing acupuncture or herbal medicine into more specific modalities, warrants clarification. Manual acupuncture with filiform needles remains the most extensively studied and clinically applied method for chronic insomnia, suitable for the vast majority of patients regardless of syndrome differentiation (15). Other techniques such as moxibustion are primarily indicated for insomnia of the deficiency-cold type and thus have a more limited scope of application (82). The included studies employed manual acupuncture, electroacupuncture, warm needle acupuncture, and auricular acupuncture (83). Although these modalities differ in the form of adjunct stimulation, their common therapeutic core is the insertion of filiform needles into specific acupoints. Electrical or thermal stimulation is applied only after needling and does not alter the primary mechanical stimulation pathway. Techniques without needling, such as pure moxibustion, were excluded because they rely predominantly on thermal effects and have a more restricted indication pattern. Thus, all needling-based acupuncture methods were merged to form a clinically coherent comparator, reflecting real-world practice where filiform needle insertion is the central intervention. Similarly, Chinese herbal medicine was analyzed as a whole because its practice is inherently based on individualized syndrome differentiation, making it methodologically challenging to isolate a single prescription.

### 4.1 Interpretation of therapeutic findings and potential mechanism

The SUCRA-based rankings indicated that combination therapy and acupuncture were associated with the highest probabilities of being the most effective interventions for both total efficacy and PSQI scores, followed by Chinese herbal medicine, with Western medication ranking last. This finding raises two clinically relevant questions: why acupuncture appears to outperform standalone herbal medicine, and what mechanisms may underlie the apparent superiority of combination therapy. This overall pattern is consistent with a previous network meta-analysis of acupuncture-related therapies for primary insomnia, which likewise found needling-based therapies to outperform conventional medication (84).

A plausible explanation may be that acupuncture and Chinese herbal medicine operate through fundamentally distinct yet potentially complementary pathways. Acupuncture is primarily a neuroregulatory therapy (85). The core acupoints frequently needled for chronic insomnia, often located on the head (e.g., Baihui GV20, Sishencong EX-HN1), hands (e.g., Shenmen HT7, Neiguan PC6), and feet (e.g., Sanyinjiao SP6), are situated in areas with rich peripheral nerve innervation (86, 87). Stimulation of these points is believed to rapidly modulate central nervous system activity (88). A growing body of evidence suggests that acupuncture may promptly influence the release of sleep-related neurotransmitters and hormones, including gamma-aminobutyric acid (GABA), melatonin, and serotonin (5-HT), while also regulating the activity of the hypothalamic-pituitary-adrenal (HPA) axis (89). Consistent with this, clinical evidence indicates that acupuncture can increase nocturnal melatonin secretion and reduce insomnia severity (90). This direct neural pathway may offer a relatively rapid mechanism for reducing hyperarousal and promoting sleep onset and maintenance. In contrast, Chinese herbal medicine, typically administered orally as multi-herb formulas such as Suanzaoren Tang or Guipi Tang, is thought to exert its effects primarily through metabolic pathways (91). After ingestion, the bioactive compounds undergo absorption, distribution, and metabolism, potentially modulating neurotransmitter systems, inflammatory cytokines, and neuroendocrine pathways such as the HPA axis over a more sustained period (92). This distinction in the routes of signal transmission, one primarily neural and the other primarily humoral and metabolic, may partly explain why acupuncture, with its more direct and rapid influence on the central nervous system, might demonstrate greater clinical efficacy in improving subjective sleep quality and overall response rates within the typical treatment durations observed in the included trials. Notably, while the metabolic pathway of herbal medicine may be slower, its multi-target actions could contribute to a more gradual and persistent rebalancing of the body's internal environment (93). This interpretation is supported by previous systematic reviews and rigorously designed randomized trials demonstrating that acupuncture significantly improves sleep quality and clinical response rates in patients with insomnia (94–96). In support of this metabolic-pathway account, a systematic review of Suanzaoren decoction, a representative herbal formula for insomnia, reported significant improvements in sleep quality compared with placebo or benzodiazepines, with fewer adverse effects (97).

Regarding the superior performance of combination therapy, several hypothetical mechanisms of synergy warrant consideration. It is possible that acupuncture acutely regulates central neurotransmitters to facilitate the rapid induction of sleep and enhancement of deep sleep, while herbal medicine acts over a longer timeframe to restore homeostatic balance, maintain sleep throughout the night, and reduce early-morning awakenings (98–101). Furthermore, acupuncture is known to modulate cerebral blood flow and may potentially enhance the blood-brain barrier permeability or cerebral delivery of certain active herbal compounds, thereby amplifying their central effects (102, 103). Conversely, herbal medicine may create a more favorable internal milieu—such as replenishing qi and blood, soothing the liver, or nourishing the heart and spleen—which enhances the body's responsiveness to acupuncture stimulation, an effect that could be described as herbal medicine potentiating the acupuncture response (104). These pathways do not necessarily conflict; rather, the combination may achieve a bidirectional and synergistic enhancement where acupuncture potentiates the central actions of herbal medicine while herbal medicine primes the body for a more robust and sustained response to acupuncture, ultimately resulting in the highest probability of clinical benefit. It must be emphasized that the proposed synergistic mechanisms are largely hypothetical and derived from preclinical or indirect clinical evidence. No direct human studies have confirmed that acupuncture enhances blood-brain barrier permeability for specific herbal compounds in chronic insomnia. Future research using factorial or mediation design is needed to delineate the exact nature of the interaction.

### 4.2 Safety and adverse events

With respect to adverse events, the SUCRA rankings showed that acupuncture had the highest probability of being the safest intervention, followed by herbal medicine, combination therapy, and Western medication. The higher incidence of adverse events associated with Western pharmacotherapy, such as daytime drowsiness, cognitive impairment, dependence, and withdrawal symptoms, has been well documented (105, 106). In the trials included in this network meta-analysis, acupuncture-related adverse events such as mild subcutaneous bleeding, bruising, or transient pain at the needling site were reported, but serious adverse events were rare (27, 45, 61, 70, 76, 77). This favorable safety profile may be explained by the fact that for chronic insomnia, the selected acupoints are predominantly located on the head and distal limbs, avoiding regions that could present risks to major organs, blood vessels, or nerves. Additionally, the acupuncture techniques employed are generally mild-to-moderate in stimulation and do not involve highly invasive or intensely stimulating procedures, thereby contributing to a low incidence of adverse reactions (107, 108).

### 4.3 Clinical implications and interpretation of the evidence

Although not all pairwise comparisons reached statistical significance, combination therapy significantly outperformed Western medication and herbal medicine alone in total efficacy, and the ranking trends consistently favored acupuncture and combination therapy across outcomes. Specifically, combination therapy consistently ranked first for total efficacy, while acupuncture alone ranked first for improving PSQI scores and minimizing adverse events. This suggests that combination therapy represents the most promising option for maximizing overall therapeutic response, whereas acupuncture alone appears particularly beneficial when the primary goal is to improve sleep quality with the lowest possible risk of side effects. The slight differences in ranking between total efficacy and PSQI may be attributable to the fact that PSQI is a multi-dimensional instrument covering subjective sleep quality, sleep latency, sleep duration, efficiency, disturbances, and daytime dysfunction; subtle variations in how each intervention modulates these specific domains could influence the overall PSQI score and ranking order. Furthermore, the data indicate that although combination therapy may be associated with a slightly higher adverse event probability than acupuncture alone—likely because the addition of herbal medicine introduces another potential source of side effects—it remains substantially safer than Western medication.

The observed superiority of combination therapy for total efficacy is consistent with the complementary neuroregulatory and metabolic mechanisms discussed above (Section 4.1); however, whether the interaction is additive or truly synergistic cannot be differentiated by the current data.

### 4.4 Limitations and future directions

Several limitations of this network meta-analysis should be acknowledged. First, all included studies were conducted in China, which may reflect differences in cultural context and medical practice and could therefore limit the generalizability of the findings to other populations. Second, the quality of the included trials varied and many did not report adequate details on randomization procedures or allocation concealment, and none successfully implemented blinding—a common challenge in acupuncture research. Third, although meta-regression identified sample size as the primary source of heterogeneity for PSQI (reducing I^2^ from 96% to 0.5%) and publication year as a significant moderator for total efficacy, residual unexplained heterogeneity may still exist due to differences in acupuncture point selection, herbal formula composition, treatment frequency, and duration among studies. Fourth, all interventions were compared over relatively short treatment periods, and long-term follow-up data were absent from the majority of included trials, preventing evaluation of the durability of treatment effects. Fifth, the acupuncture therapy node included manual acupuncture, electroacupuncture, warm needle acupuncture, and auricular acupuncture. Although these techniques vary in adjunct stimulation, they share needle insertion as their common therapeutic core. Finer subdivision would have produced an extremely sparse evidence network, compromising the stability of the analysis. This grouping may introduce some clinical heterogeneity, and future studies with more primary data could compare specific acupuncture modalities. Finally, the precise nature and direction of the interaction between acupuncture and herbal medicine in combined therapy could not be determined. In addition, trials comparing the eligible interventions solely against sham or placebo control were excluded per the pre-specified protocol; consequently, the relative effects estimated here serve as benchmarks among active treatments only, and absolute effects vs. inert controls could not be evaluated within this network. Furthermore, adverse-event reporting was not standardized—none of the AE-reporting trials described prospective, protocol-defined monitoring—which precluded a sensitivity analysis restricted to trials with standardized adverse-event ascertainment; the safety results should therefore be interpreted with caution. Finally, no formal GRADE or CINeMA assessment of the certainty of the evidence was performed; considering the risk of bias in the included trials, the substantial heterogeneity for PSQI, and the single-country evidence base, the overall certainty of the evidence should be regarded as low to very low. Moreover, node-splitting detected statistically significant inconsistency between direct and indirect evidence for two comparisons of total efficacy (*P* = 0.022 and *P* = 0.008), which may reflect differences in trial quality, treatment duration, and implementation of efficacy grading thresholds across comparisons; the corresponding TE estimates should be interpreted with caution.

Future research should aim to address these gaps. Rigorously designed, multicenter, large-scale RCTs are warranted to directly compare acupuncture, herbal medicine, and their combination, ideally incorporating both subjective and objective sleep outcomes such as actigraphy and polysomnography, as well as long-term follow-up to assess sustained benefits and safety. Stratified analyses based on traditional Chinese medicine syndrome differentiation may help identify subgroups of patients who derive the greatest benefit from each modality. Finally, and importantly, mechanistic studies—both clinical experimental and preclinical—are urgently needed to elucidate the precise nature of the acupuncture-herb interaction underlying the synergistic effect observed with combination therapy. Specifically, future research should investigate whether acupuncture enhances the pharmacodynamic profile of herbal medicine, whether herbal medicine potentiates the neurophysiological response to acupuncture, or whether a bidirectional synergistic mechanism is at play. Clarifying this question will not only inform clinical decision-making but also provide a scientific basis for optimizing integrated traditional Chinese medicine treatment strategies for chronic insomnia.

## 5 Conclusion

This network meta-analysis of 59 RCTs provides a comprehensive comparison of acupuncture, Chinese herbal medicine, combined acupuncture-herbal therapy, and Western medication for adult chronic insomnia. Combined acupuncture-herbal therapy showed the highest probability of being the most effective intervention for total efficacy and significantly outperformed Western medication and herbal medicine alone, although no significant difference was observed compared with acupuncture alone. Acupuncture monotherapy ranked first for improving sleep quality (PSQI) and demonstrated the most favorable safety profile. The observed superiority of combination therapy may reflect a complementary, potentially synergistic interaction—whereby acupuncture provides rapid neuroregulatory modulation of sleep-related central pathways while herbal medicine exerts sustained metabolic and humoral regulation—although this hypothesis remains speculative and requires confirmation in mechanistic and factorial-design studies. From a clinical practice perspective, these findings provide an evidence-based hierarchy to guide treatment selection for adults with chronic insomnia. For patients seeking to maximize the overall therapeutic response—particularly those with an inadequate response to, or intolerance of, hypnotic medications—combined acupuncture-herbal therapy may be considered a preferred option. For patients in whom safety and tolerability are the primary concerns, such as older adults or those at risk of drug dependence, acupuncture monotherapy represents a favorable alternative with the lowest probability of adverse events. Given the limited accessibility of cognitive behavioral therapy for insomnia and the well-documented drawbacks of long-term hypnotic use, these results support the integration of acupuncture-based traditional Chinese medicine modalities into the stepped-care management of chronic insomnia and provide clinicians and patients with a comparative evidence base for shared decision-making. Given the moderate-to-low methodological quality of the included trials, the absence of blinding, and the limited geographical representation, these findings should be interpreted with caution. High-quality, multicenter RCTs incorporating objective sleep outcomes and long-term follow-up are warranted to validate these results.

## Data Availability

The original contributions presented in the study are included in the article/Supplementary Material, further inquiries can be directed to the corresponding author/s.

## References

[1] Morin CM Benca R. Chronic insomnia. Lancet. (2012) 379(9821):1129–41. 10.1016/S0140-6736(11)60750-2 22265700

[2] Matheson EM Brown BD DeCastro AO. Treatment of chronic insomnia in adults. Am Fam Physician. (2024) 109(2):154–60. 38393799

[3] Sutton EL. Insomnia. Med Clin North Am. (2014) 98(3):565–81. 10.1016/j.mcna.2014.01.008 24758961

[4] Morin CM Jarrin DC. Epidemiology of insomnia: prevalence, course, risk factors, and public health burden. Sleep Med Clin. (2022) 17(2):173–91. 10.1016/j.jsmc.2022.03.003 35659072

[5] Riemann D Spiegelhalder K Feige B Voderholzer U Berger M Perlis M. The neurobiology, investigation, and treatment of chronic insomnia. Lancet Neurol. (2015) 14(5):547–58. 10.1016/S1474-4422(15)00021-6 25895933

[6] Levenson JC Kay DB Buysse DJ. The pathophysiology of insomnia. Chest. (2015) 147(4):1179–92. 10.1378/chest.14-1617 25846534 PMC4388122

[7] Winkelman JW Buxton OM Jensen JE Benson KL O'Connor SP Wang W. Reduced brain GABA in primary insomnia: preliminary data from 4 T proton magnetic resonance spectroscopy (1H-MRS). Sleep. (2008) 31(11):1499–506. 10.1093/sleep/31.11.1499 19014069 PMC2579978

[8] Kaufman MJ Meloni EG. Xenon gas as a potential treatment for opioid use disorder, alcohol use disorder, and related disorders. Med Gas Res. (2025) 15(2):234–53. 10.4103/mgr.MEDGASRES-D-24-00063 39812023 PMC11918480

[9] Li XR Cui JJ Ge WP Wang ZW Chu YC Zheng GR. Ozonated autohemotherapy combined with pulsed radiofrequency in the treatment of thoracic postherpetic neuralgia in older adults: a retrospective study. Med Gas Res. (2024) 14(1):12–8. 10.4103/2045-9912.372666 37721250 PMC10710284

[10] Cheng J Williams JP Zhou L Wang P-C Sun L-N Li R-H. Ozone rectal insufflation mitigates chronic rapid eye movement sleep deprivation-induced cognitive impairment through inflammation alleviation and gut microbiota regulation in mice. Med Gas Res. (2024) 14(4):213–24. 10.4103/mgr.MEDGASRES-D-23-00036 39073330 PMC11257187

[11] Chan NY Chan JWY Li SX Wing YK. Non-pharmacological approaches for management of insomnia. Neurotherapeutics. (2021) 18(1):32–43. 10.1007/s13311-021-01029-2 33821446 PMC8116473

[12] Palagini L Brugnoli R Dell’ Osso BM Di Nicola M Maina G Martinotti G. Clinical practice guidelines for switching or deprescribing hypnotic medications for chronic insomnia: results of European neuropsychopharmacology and sleep expert’s consensus group. Sleep Med. (2025) 128:117–26. 10.1016/j.sleep.2025.01.033 39923608

[13] Kay-Stacey M Attarian H. Advances in the management of chronic insomnia. Bmj-British Medical Journal. (2016) 354:i2123. 10.1136/bmj.i2123 27383400

[14] Ye Z Lai H Ning J Liu J Huang J Yang S. Traditional Chinese medicine for insomnia: recommendation mapping of the global clinical guidelines. J Ethnopharmacol. (2024) 322:117601. 10.1016/j.jep.2023.117601 38122913

[15] Zhao FY Spencer SJ Kennedy GA Zheng Z Conduit R Zhang W-J. Acupuncture for primary insomnia: effectiveness, safety, mechanisms and recommendations for clinical practice. Sleep Med Rev. (2024) 74:101892. 10.1016/j.smrv.2023.101892 38232645

[16] Liu X Sun P Bao X Cao Y Wang L Wang Q. Potential mechanisms of traditional Chinese medicine in treating insomnia: a network pharmacology, GEO validation, and molecular-docking study. Medicine (Baltimore). (2024) 103(18):e38052. 10.1097/MD.0000000000038052 38701256 PMC11062677

[17] Cheuk DK Yeung WF Chung KF Wong V. Acupuncture for insomnia. Cochrane Database Syst Rev. (2012) 2012(9):Cd005472. 10.1002/14651858.CD005472.pub3 22972087 PMC11262418

[18] Wang J Zhao H Shi K Wang M. Treatment of insomnia based on the mechanism of pathophysiology by acupuncture combined with herbal medicine: a review. Medicine (Baltimore). (2023) 102(11):e33213. 10.1097/MD.0000000000033213 36930068 PMC10019201

[19] Li Z Yin S Feng J Gao X Yang Q Zhu F. Acupuncture combined with Chinese herbal medicine in the treatment of perimenopausal insomnia: a systematic review and meta-analysis. Medicine (Baltimore). (2023) 102(45):e35942. 10.1097/MD.0000000000035942 37960761 PMC10637479

[20] Page MJ McKenzie JE Bossuyt PM Boutron I Hoffmann TC Mulrow CD. The PRISMA 2020 statement: an updated guideline for reporting systematic reviews. Br Med J. (2021) 372:n71. 10.1136/bmj.n71 PMC8005924 33782057

[21] Sateia MJ. International classification of sleep disorders-third edition: highlights and modifications. Chest. (2014) 146(5):1387–94. 10.1378/chest.14-0970 25367475

[22] Sterne JAC Savović J Page MJ Elbers RG Blencowe NS Boutron I. Rob 2: a revised tool for assessing risk of bias in randomised trials. Br Med J. (2019) 366:l4898. 10.1136/bmj.l4898 31462531

[23] An Q Zhong L. Zuogui pill for refractory insomnia: a randomized parallel controlled study. J Pract Tradit Chin Intern Med. (2015) 29(2):74–6.

[24] Ba L. Effect of acupuncture combined with guipi decoction on sleep quality in patients with insomnia of heart-spleen deficiency. Guide China Med. (2023) 21(9):118–20.

[25] Chen G Pan H. Clinical observation on guipi decoction combined with acupuncture for 38 cases of insomnia of heart-spleen deficiency type. Chin J Ethnomed Ethnopharm. (2020) 29(15):97–9.

[26] Chen X Xu K Qin X. Observation on the therapeutic effect of electroacupuncture for female perimenopausal insomnia. Shanghai J Acupunct Moxibustion. (2011) 30(6):366–7.

[27] Cui Y. Effect of acupuncture plus auricular point sticking combined with Chinese herbal medicine on intractable insomnia. Chin J Urban Rural Enterp Health. (2021) 36(10):126–7.

[28] Fan J Xiong J He X. Clinical study of yangxin hewei yimian decoction combined with acupuncture at anmian point for senile insomnia. Chin Arch Tradit Chin Med. (2019) 37(2):466–9.

[29] Guo D Wu Q. Acupuncture combined with modified xiaochaihu decoction for 20 cases of liver-qi stagnation insomnia. Res Tradit Chin Med. (2020) 33(5):50–2.

[30] Hao J. Clinical observation on jieyu anshen decoction for insomnia in menopausal women. Chin Arch Tradit Chin Med. (2011) 29(7):1691–3.

[31] He X Xu H. Observation on the therapeutic effect of acupuncture combined with medicine in the treatment of insomnia. J Chin Med Mater. (2006) 29(2):203–4.

[32] Hu J Tian H. Effect of spirit-regulating acupuncture on sleep quality, TCM syndrome scores and SDS scores in patients with insomnia of yin deficiency with effulgent fire type. J Sichuan Tradit Chin Med. (2021) 39(11):211–4.

[33] Hu L Wang J. Clinical effect of electroacupuncture at jiaji points for insomnia with depressive disorder analyzed by resting-state functional MRI. Pract Clin J Integr Tradit Chin West Med. (2024) 24(1):56–9.

[34] Huang L Zhang Y. Clinical study on acupuncture combined with medicine in treating menopausal insomnia of yin deficiency and fire hyperactivity type. Guangming J Chin Med. (2023) 38(16):3088–91.

[35] Huang X Wang P. Clinical observation of eye acupuncture for intractable insomnia. Liaoning J Tradit Chin Med. (2010) 37(9):1801–2.

[36] Ji X Wang Q Zhu W. Effect of acupuncture on the expression of different neurotransmitters in patients with liver-depression-fire type insomnia. Chin Acupunct Moxibustion. (2015) 35(6):549–52. 26480548

[37] Jia H Wang C Xin H Li J Liang X Duo H. Clinical efficacy evaluation of self-made anshen yangxue decoction combined with warm needling and auricular acupoint therapy for refractory insomnia. J Hebei Tradit Chin Med Pharmacol. (2021) 36(1):43–6.

[38] Jiang X Chen J Zhu S. A randomized controlled trial on the efficacy and safety of mind-body regulating acupuncture for chronic insomnia. Smart Healthc. (2022) 8(33):178–81.

[39] Jin Y Hai Y. Acupuncture combined with guipi decoction for heart-spleen deficiency type insomnia: a randomized parallel controlled study. J Pract Tradit Chin Intern Med. (2016) 30(6):84–7.

[40] Li D Zhang G Yue Y. Clinical efficacy observation on baihe dihuang decoction combined with acupuncture for insomnia of yin deficiency constitution. Inner Mongolia J Tradit Chin Med. (2024) 43(2):11–3.

[41] Li F Ren Y An S Wang D. Clinical observation of acupuncture at jiaji (EX-B2) points for insomnia. J Clin Acupunct Moxib. (2014) 30(3):16–8.

[42] Li P Ding N Xiang S. Clinical observation of yin-yang harmonization therapy for insomnia of heart-kidney disharmony type. Shanxi J Tradit Chin Med. (2017) 33(8):12–3.

[43] Li P Liu X. Clinical observation on tongdu tiaoshen acupuncture for insomnia. J Clin Acupunct Moxibustion. (2013) 29(4):17–8.

[44] Liao X. Randomized parallel controlled study of bo zhiyun’s abdominal acupuncture combined with qiju dihuang decoction for insomnia of liver-kidney yin deficiency in middle-aged and elderly patients. J Pract Tradit Chin Intern Med. (2013) 27(2):120–1.

[45] Lin G. Clinical effect of traditional Chinese medicine combined with acupuncture and auricular point pressing with beans in the treatment of insomnia. Nei Mongol J Tradit Chin Med. (2021) 40(1):115–6.

[46] Liu J. Acupuncture combined with ningshen decoction no. 2 for liver-fire disturbing the heart type insomnia: a randomized parallel controlled study. J Pract Tradit Chin Intern Med. (2014) 28(11):129–31.

[47] Liu Q. Clinical study on acupuncture combined with yangxin anshen decoction in treating insomnia of heart-spleen deficiency type. Inner Mongol J Tradit Chin Med. (2019) 38(6):101–2.

[48] Liu Q Mao R Hu Q Huang Y. Modified xiaoyao powder combined with acupuncture for menstrual insomnia of liver depression and spleen deficiency type. Nei Mongol J Tradit Chin Med. (2025) 44(2):72–3,99.

[49] Liu Z Niu W. Scalp hairline zone row needling for 160 cases of insomnia. Shaanxi J Tradit Chin Med. (2007) 28(6):716–7.

[50] Ma X Deng L Kuang J Ou W Cao Y. Observation on the effect of acupuncture on the ren meridian for insomnia. Guide China Med. (2013) 11(36):198–9.

[51] Ma Z Li G Duan H. Observation on the efficacy of acupuncture for insomnia in 31 cases. Guangming J Chin Med. (2006) 21(4):25–6.

[52] Ni J Zhu W. Clinical comparative study of scalp point penetration needling as main therapy for 76 cases of insomnia. J Clin Acupunct Moxibustion. (2006) 22(12):35–6.

[53] Pan Q Zhang L Yang J He Y. Analysis of the efficacy of acupuncture for intractable insomnia. Chin Trop Med. (2005) 5(8):1705.

[54] Pi Y Tang Y Wang Z Wang L. Effect of acupuncture treatment for primary insomnia on each factor of the Pittsburgh sleep quality index. J Sichuan Tradit Chin Med. (2017) 35(11):188–91.

[55] Tan Z. Clinical observation on yin-zhong-yin-yang acupuncture method for senile insomnia. Chin Manip Rehabil Med. (2020) 11(19):25–6.

[56] Tian C Wang L Wang S Zou T Liu Z Li S. A randomized controlled trial of hewei anshen decoction for 52 male patients with stress insomnia. J Tradit Chin Med. (2024) 65(22):2326–31.

[57] WANG R ZHANG N- TAN Q- YANG D- GUAN L YANG Z- Clinical observation on electroacupuncture combined with Chinese herbal medicine for insomnia. J Clin Acupunct Moxibustion. (2006) 22(5):34–5. 10.1016/S1003-5257(12)60025-7

[58] Wang L. Randomized parallel controlled study on direct acupuncture combined with ningshen decoction in treating insomnia of liver fire disturbing heart type. J Pract Tradit Chin Intern Med. (2013) 27(4):114–5.

[59] Wang W. Clinical observation on the therapeutic effect of acupuncture in treating insomnia. Mod Diagn Treat. (2013) 24(6):1285–6.

[60] Wang X. Observation on the therapeutic effect of sedation and tranquilization acupuncture in 63 patients with insomnia. Shanghai J Acupunct Moxibustion. (2005) 24(6):21–2.

[61] Wang Y Zhang L Han Y Li P. Observation on the therapeutic effect of du-regulating and mind-tranquilizing acupuncture for insomnia. J Acupunct Tuina Sci. (2016) 14(4):274–8. 10.1007/s11726-016-0935-1

[62] Wang Y Zhang R Wang C Hu H Xiao M Sui R. Acupuncture at dong’s extraordinary points combined with sini powder for liver-depression and spleen-deficiency type insomnia in the elderly: a clinical observation. Chin J Geriatr Care. (2021) 19(3):17–9.

[63] Wu H. Modified guipi decoction combined with body acupuncture for 48 cases of insomnia of heart-spleen deficiency type. J Tradit Chin Med Res. (2014) 27(4):51–2.

[64] Xu D Gao H. Clinical observation of ganmai dazao decoction combined with suanzaoren decoction for insomnia of yin deficiency with fire hyperactivity type. Guangming J Chin Med. (2020) 35(21):3376–8.

[65] Xu H Hu Z Wang Q. Clinical study on acupuncture combined with medicine in the treatment of insomnia. Liaoning J Tradit Chin Med. (2003) 30(7):570–1.

[66] Xu H Wang P He W. Clinical observation of eye acupuncture therapy for insomnia. J Liaoning Univ Tradit Chin Med. (2014) 16(8):109–11.

[67] Xu S. Wendan anmian decoction combined with acupuncture for insomnia of phlegm-heat disturbing the heart type: a randomized parallel controlled study. J Pract Tradit Chin Intern Med. (2015) 29(5):156–7.

[68] Xu Y Lin X. Clinical study of acupuncture at six spirit-regulating points combined with three sleep points for primary insomnia of liver-fire disturbing heart type. J New Chin Med. (2020) 52(3):151–3.

[69] Yang X. Efficacy observation of yueju pill combined with superficial acupuncture for perimenopausal insomnia. Guangming J Chin Med. (2017) 32(1):84–5.

[70] Yao W Li D Wu Q Yan J Ye H. Clinical observation on mind-regulating acupuncture with extraordinary points for insomnia. Clin J Tradit Chin Med. (2014) 6(04):4–6.

[71] Ye X Dai W. Observation on the therapeutic effect of acupuncture combined with medicine in the treatment of insomnia. J Yunnan Coll Tradit Chin Med. (2006) 29(4):26–7.

[72] Yin L. Eryin decoction for diabetic insomnia: a randomized parallel controlled study. J Pract Tradit Chin Intern Med. (2013) 27(6):16–8.

[73] Zhang Q. Clinical observation on liver-regulating and spleen-harmonizing Chinese herbal medicine for 36 cases of perimenopausal insomnia. J Tradit Chin Med. (2006) 47(9):678–80.

[74] Zhang S Tian W. Randomized parallel controlled study of tian weizhu’s eye acupuncture combined with wendan decoction for insomnia. J Pract Tradit Chin Intern Med. (2013) 27(12):24–5.

[75] Zhang T. Acupuncture and moxibustion combined with Chinese herbal medicine in treating 36 cases of insomnia of heart-spleen deficiency type. J Extern Ther Tradit Chin Med. (2011) 20(1):26–7.

[76] Zhang Z Liu Z. Clinical observation on the therapeutic effect of acupuncture on insomnia patients and its influence on the levels of inflammatory factors. Shaanxi J Tradit Chin Med. (2015) 36(10):1420–2.

[77] Zhong L. Observation on the therapeutic effect of traditional Chinese medicine acupuncture in patients with insomnia. World J Sleep Med. (2019) 6(8):1066–7.

[78] Zhu J Liu T Hu F Sheng J Jin Y. Acupuncture for perimenopausal early-awakening insomnia: a randomized controlled trial. Chin Acupunct Moxibustion. (2022) 42(6):608–12. 10.13703/j.0255-2930.20211009-k0004 35712942

[79] Zhu X Zeng H Zhu S Zhu S. Clinical observation of fuzi decoction combined with guizhi gancao longgu muli decoction for insomnia of heart-kidney yang deficiency type. Chin Arch Tradit Chin Med. (2025) 43(11):200–3.

[80] Zhu X Zhang F Dai J Dou Z Yang M Zhu R. Clinical study on tongdu tiaoshen acupuncture for insomnia. Lishizhen Med Mater Med Res. (2019) 30(2):389–90.

[81] Zou Y. Clinical observation on mind-tranquilizing and will-stabilizing electroacupuncture for psychogenic insomnia. Heilongjiang J Tradit Chin Med. (2008) 37(1):40–1.

[82] Sun YJ Yuan JM Yang ZM. Effectiveness and safety of moxibustion for primary insomnia: a systematic review and meta-analysis. BMC Complement Altern Med. (2016) 16(1):217. 10.1186/s12906-016-1179-9 27411310 PMC4944240

[83] Lu G Chen F Guo C Wu J. Acupuncture for senile insomnia: a systematic review of acupuncture point. Arch Gerontol Geriatr. (2024) 127:105586. 10.1016/j.archger.2024.105586 39096556

[84] Lu Y Zhu H Wang Q Tian C Lai H Hou L. Comparative effectiveness of multiple acupuncture therapies for primary insomnia: a systematic review and network meta-analysis of randomized trial. Sleep Med. (2022) 93:39–48. 10.1016/j.sleep.2022.03.012 35405419

[85] Zhuang Y Xing JJ Li J Zeng BY Liang FR. History of acupuncture research. Int Rev Neurobiol. (2013) 111:1–23. 10.1016/B978-0-12-411545-3.00001-8 24215915

[86] Lina Q Yinan S Lianhong T Yanshu J Yongsheng Y. Efficacy of electroacupuncture stimulating shenmen (HT7), baihui (GV20), sanyinjiao (SP6) on spatial learning and memory deficits in rats with insomnia induced by para-chlorophenylalanine: a single acupoint combined acupoints. J Tradit Chin Med. (2023) 43(4):704–14. 10.19852/j.cnki.jtcm.20230308.001 37454255 PMC10320443

[87] Wu D Liu XN Jia X Ha LJ. Effect of single acupoint and acupoints compatibility on PSQI in patients with primary insomnia: a randomized controlled clinical trial. Medicine (Madr). (2025) 104(48):e46151. 10.1097/MD.0000000000046151 PMC12662494 41327705

[88] Zhao K. Acupuncture for the treatment of insomnia. Int Rev Neurobiol. (2013) 111:217–34. 10.1016/B978-0-12-411545-3.00011-0 24215925

[89] Yao L Liu Y Li M Zheng H Sun M He M. The central regulatory effects of acupuncture in treating primary insomnia: a review. Front Neurol. (2024) 15:1406485. 10.3389/fneur.2024.1406485 39719980 PMC11666528

[90] Spence DW Kayumov L Chen A Lowe A Jain U Katzman MA. Acupuncture increases nocturnal melatonin secretion and reduces insomnia and anxiety: a preliminary report. J Neuropsychiatry Clin Neurosci. (2004) 16(1):19–28. 10.1176/jnp.16.1.19 14990755

[91] Wang M Chen L Liu D Chen H Tang DD Zhao YY. Metabolomics highlights pharmacological bioactivity and biochemical mechanism of traditional Chinese medicine. Chem Biol Interact. (2017) 273:133–41. 10.1016/j.cbi.2017.06.011 28619388

[92] Zhou J Guo X Yu W Bu H Du Y Li S. Pharmacological action, mechanism and structure-activity relationship of traditional Chinese medicine polysaccharide in the treatment of Alzheimer’s disease: a review. Int J Biol Macromol. (2025) 330(Pt 2):148017. 10.1016/j.ijbiomac.2025.148017 41038475

[93] Zhao X Li Q Bian L Zheng X Zheng J Zhang Y. Using immobilized G-protein coupled receptors to screen bioactive traditional Chinese medicine compounds with multiple targets. J Pharm Biomed Anal. (2012) 70:549–52. 10.1016/j.jpba.2012.05.004 22651959

[94] Yin X Gou M Xu J Dong B Yin P Masquelin F. Efficacy and safety of acupuncture treatment on primary insomnia: a randomized controlled trial. Sleep Med. (2017) 37:193–200. 10.1016/j.sleep.2017.02.012 28899535

[95] Cao H Pan X Li H Liu J. Acupuncture for treatment of insomnia: a systematic review of randomized controlled trials. J Altern Complement Med. (2009) 15(11):1171–86. 10.1089/acm.2009.0041 19922248 PMC3156618

[96] Huang W Kutner N Bliwise DL. A systematic review of the effects of acupuncture in treating insomnia. Sleep Med Rev. (2009) 13(1):73–104. 10.1016/j.smrv.2008.04.002 19097814

[97] Xie C- Gu Y Wang W-W Lu L Fu D- Liu A- Efficacy and safety of suanzaoren decoction for primary insomnia: a systematic review of randomized controlled trials. BMC Complement Altern Med. (2013) 13:18. 10.1186/1472-6882-13-18 23336848 PMC3554563

[98] Han JS Tang J Ren MF Zhou ZF Fan SG Qiu XC. Central neurotransmitters and acupuncture analgesia. Am J Chin Med. (1980) 8(4):331–48. 10.1142/S0192415X80000311 6113756

[99] Omura Y. Connections found between each meridian (heart, stomach, triple burner, etc.) & organ representation area of corresponding internal organs in each side of the cerebral cortex; release of common neurotransmitters and hormones unique to each meridian and corresponding acupuncture point & internal organ after acupuncture, electrical stimulation, mechanical stimulation (including shiatsu), soft laser stimulation or QI gong. Acupunct Electrother Res. (1989) 14(2):155–86. 10.3727/036012989816358470 2575347

[100] Soligo M Nori SL Protto V Florenzano F Manni L. Acupuncture and neurotrophin modulation. Int Rev Neurobiol. (2013) 111:91–124. 10.1016/B978-0-12-411545-3.00005-5 24215919

[101] Yang Y Wu Y Xu P Guo F Guo F Yang B. Nyctinastic herbs decoction improves para-chlorophenylalanine-induced insomnia by regulating the expression level of neurotransmitters. Ann Transl Med. (2021) 9(20):1524. 10.21037/atm-21-4462 34790730 PMC8576665

[102] Zhang C Zheng J Yu X Kuang B Dai X Zheng L. Baihui” (DU20)-penetrating “qubin” (GB7) acupuncture on blood-brain barrier integrity in rat intracerebral hemorrhage models via the RhoA/ROCK II/MLC 2 signaling pathway. Animal Model Exp Med. (2024) 7(5):740–57. 10.1002/ame2.12374 38379356 PMC11528382

[103] Zhang ZH Gu Y Huang Z Liu XY Xu WT Zhang XC. Acupuncture regulates astrocyte neurotoxic polarization to protect blood-brain barrier integrity in delayed thrombolysis through mediating ERK1/2/Cx43 axis. IBRO Neurosci Rep. (2025) 18:604–18. 10.1016/j.ibneur.2025.04.005 40292080 PMC12022658

[104] Li W Wang Q Dang C Xiong Y Lu Y. Chinese herbal medicine for insomnia: a systematic review and network meta-analysis. Medicine (Baltimore). (2025) 104(33):e43789. 10.1097/MD.0000000000043789 40826721 PMC12366933

[105] Lader M. Benzodiazepines revisited–will we ever learn? Addiction. (2011) 106(12):2086–109. 10.1111/j.1360-0443.2011.03563.x 21714826

[106] Dündar Y Boland A Strobl J Dodd S Haycox A Bagust A. Newer hypnotic drugs for the short-term management of insomnia: a systematic review and economic evaluation. Health Technol Assess. (2004) 8(24):iii–x;1–125. 10.3310/hta8240 15193209

[107] Yoon DE Lee IS Chae Y. Identifying dose components of manual acupuncture to determine the dose-response relationship of acupuncture treatment: a systematic review. Am J Chin Med. (2022) 50(3):653–71. 10.1142/S0192415X22500264 35300569

[108] White A Cummings M Barlas P Cardini F Filshie J Foster NE. Defining an adequate dose of acupuncture using a neurophysiological approach–a narrative review of the literature. Acupunct Med. (2008) 26(2):111–20. 10.1136/aim.26.2.111 18591910

