# Supplementary material for: Efficacy of acupuncture, Chinese herbal medicine, combined acupuncture-herbal therapy, and western medication for adult chronic insomnia: a network meta-analysis
Source: Front Med (Lausanne). 2026 Sep 30;13:1961539. doi: 10.3389/fmed.2026.1961539 (PMC13661500; doi:10.3389/fmed.2026.1961539)
Supplement: Supplementary file 1 [file Supplementaryfile1.docx]

****Appendix 1. The search strategy****

**The search strategy (Pubmed)**

| Search number | Query | Results |
| --- | --- | --- |
| 1 | ( "chronic insomnia"[Title/Abstract] OR "chronic insomnia disorder"[Title/Abstract] OR "primary insomnia"[Title/Abstract] OR "persistent insomnia"[Title/Abstract] OR ("insomnia disorder"[Title/Abstract] AND chronic[Title/Abstract]) OR (insomnia[Title/Abstract] AND chronic[Title/Abstract]) ) AND ( "Acupuncture Therapy"[Mesh] OR acupuncture[Title/Abstract] OR "manual acupuncture"[Title/Abstract] OR electroacupuncture[Title/Abstract] OR "electro-acupuncture"[Title/Abstract] OR "Medicine, Chinese Traditional"[Mesh] OR "Drugs, Chinese Herbal"[Mesh] OR "Chinese herbal medicine"[Title/Abstract] OR "traditional Chinese medicine"[Title/Abstract] OR "Chinese medicine"[Title/Abstract] OR decoction[Title/Abstract] OR "Hypnotics and Sedatives"[Mesh] OR hypnotic*[Title/Abstract] OR benzodiazepine*[Title/Abstract] OR zolpidem[Title/Abstract] OR zopiclone[Title/Abstract] OR eszopiclone[Title/Abstract] OR zaleplon[Title/Abstract] ) AND ( randomized controlled trial[Publication Type] OR controlled clinical trial[Publication Type] OR random*[Title/Abstract] OR randomly[Title/Abstract] ) NOT ( animals[MeSH Terms] NOT humans[MeSH Terms] ) NOT ( child*[Title/Abstract] OR adolescent*[Title/Abstract] OR pediatric*[Title/Abstract] OR teen*[Title/Abstract] OR infant*[Title/Abstract] OR depression[Title/Abstract] OR depressive[Title/Abstract] OR anxiety[Title/Abstract] OR cancer[Title/Abstract] OR tumor[Title/Abstract] OR stroke[Title/Abstract] OR poststroke[Title/Abstract] OR "post-stroke"[Title/Abstract] OR menopause[Title/Abstract] OR perimenopaus*[Title/Abstract] OR pregnancy[Title/Abstract] OR pregnant[Title/Abstract] OR dialysis[Title/Abstract] OR hemodialysis[Title/Abstract] OR pain[Title/Abstract] OR parkinson*[Title/Abstract] OR dementia[Title/Abstract] OR schizophrenia[Title/Abstract] OR bipolar[Title/Abstract] OR apnea[Title/Abstract] ) | 323 |

**The search strategy (Embase)**

| Search number | Query | Results |
| --- | --- | --- |
| 1 | ('chronic insomnia':ti,ab OR 'chronic insomnia disorder':ti,ab OR 'primary insomnia':ti,ab OR 'persistent insomnia':ti,ab OR ('insomnia disorder':ti,ab AND chronic:ti,ab) OR (insomnia:ti,ab AND chronic:ti,ab)) AND ('acupuncture therapy'/exp OR 'electroacupuncture'/exp OR acupuncture:ti,ab OR 'manual acupuncture':ti,ab OR electroacupuncture:ti,ab OR 'electro-acupuncture':ti,ab OR 'traditional chinese medicine'/exp OR 'chinese herbal drug' OR 'chinese herbal medicine':ti,ab OR 'traditional chinese medicine':ti,ab OR 'chinese medicine':ti,ab OR decoction:ti,ab OR 'hypnotic sedative agent'/exp OR hypnotic*:ti,ab OR benzodiazepine*:ti,ab OR zolpidem:ti,ab OR zopiclone:ti,ab OR eszopiclone:ti,ab OR zaleplon:ti,ab) AND ('randomized controlled trial'/exp OR 'controlled clinical trial'/exp OR random*:ti,ab OR randomly:ti,ab) NOT ('animal'/exp NOT 'human'/exp) NOT (child*:ti,ab OR adolescent*:ti,ab OR pediatric*:ti,ab OR teen*:ti,ab OR infant*:ti,ab OR depression:ti,ab OR depressive:ti,ab OR anxiety:ti,ab OR cancer:ti,ab OR tumor:ti,ab OR stroke:ti,ab OR poststroke:ti,ab OR 'post-stroke':ti,ab OR menopause:ti,ab OR perimenopaus*:ti,ab OR pregnancy:ti,ab OR pregnant:ti,ab OR dialysis:ti,ab OR hemodialysis:ti,ab OR pain:ti,ab OR parkinson*:ti,ab OR dementia:ti,ab OR schizophrenia:ti,ab OR bipolar:ti,ab OR apnea:ti,ab) | 686 |

**The search strategy (web of science)**

| Search number | Query | Results |
| --- | --- | --- |
| 1 | TS=(  "chronic insomnia"  OR "chronic insomnia disorder"  OR "primary insomnia"  OR "persistent insomnia"  OR ("insomnia disorder" AND chronic)  OR (insomnia AND chronic)  )  AND TS=(  acupuncture  OR "manual acupuncture"  OR electroacupuncture  OR "electro-acupuncture"  OR "traditional Chinese medicine"  OR "Chinese herbal medicine"  OR "Chinese medicine"  OR decoction  OR hypnotic*  OR benzodiazepine*  OR zolpidem  OR zopiclone  OR eszopiclone  OR zaleplon  )  AND TS=(  random*  OR randomly  OR trial  OR placebo  )  NOT TS=(  child*  OR adolescent*  OR pediatric*  OR teen*  OR infant*  OR depression  OR depressive  OR anxiety  OR cancer  OR tumor  OR stroke  OR poststroke  OR "post-stroke"  OR menopause  OR perimenopaus*  OR pregnancy  OR pregnant  OR dialysis  OR hemodialysis  OR pain  OR parkinson*  OR dementia  OR schizophrenia  OR bipolar  OR apnea  ) | 349 |

**The search strategy (Cochrane Library)**

| Search number | Query | Results |
| --- | --- | --- |
| 1 | (  ("chronic insomnia" OR "chronic insomnia disorder" OR "primary insomnia" OR "persistent insomnia" OR ("insomnia disorder" AND chronic) OR (insomnia AND chronic)):ti,ab,kw  )  AND  (  ([mh "Acupuncture Therapy"] OR acupuncture OR "manual acupuncture" OR electroacupuncture OR "electro-acupuncture"):ti,ab,kw  OR  ([mh "Medicine, Chinese Traditional"] OR [mh "Drugs, Chinese Herbal"] OR "Chinese herbal medicine" OR "traditional Chinese medicine" OR "Chinese medicine" OR decoction):ti,ab,kw  OR  ([mh "Hypnotics and Sedatives"] OR hypnotic* OR benzodiazepine* OR zolpidem OR zopiclone OR eszopiclone OR zaleplon):ti,ab,kw  )  AND  (  random* OR randomly OR trial  ):ti,ab,kw  NOT  (  child* OR adolescent* OR pediatric* OR teen* OR infant* OR depression OR depressive OR anxiety OR cancer OR tumor OR stroke OR poststroke OR "post-stroke" OR menopause OR perimenopaus* OR pregnancy OR pregnant OR dialysis OR hemodialysis OR pain OR parkinson* OR dementia OR schizophrenia OR bipolar OR apnea  ):ti,ab,kw | 355 |

**The search strategy (中国知网)**

| Search number | Query | Results |
| --- | --- | --- |
| 1 | 慢性失眠 + 慢性失眠障碍 + 原发性失眠 + 持续性失眠 + 慢性失眠症 + 失眠症  AND  针刺 + 针灸 + 毫针 + 电针 + 中药 + 中药汤剂 + 汤剂 + 唑吡坦 + 佐匹克隆 + 右佐匹克隆 + 扎来普隆  AND  随机 + 随机对照 + 随机对照试验  NOT  儿童 + 青少年 + 婴儿 + 癌症 + 肿瘤 + 卒中 + 脑卒中 + 妊娠 + 孕妇 + 帕金森 + 痴呆 + 精神分裂症 + 双相情感障碍 + 睡眠呼吸暂停 | 353 |

**The search strategy (万方)**

| Search number | Query | Results |
| --- | --- | --- |
| 1 | (慢性失眠 OR 慢性失眠障碍 OR 慢性失眠症 OR 原发性失眠 OR 持续性失眠)  AND  (针刺 OR 针灸 OR 毫针 OR 电针 OR 中医药 OR 传统中医药 OR 中药 OR 中药汤剂 OR 汤剂 OR 西药 OR 安眠药 OR 镇静催眠药 OR 苯二氮卓类 OR 非苯二氮卓类 OR 唑吡坦 OR 佐匹克隆 OR 右佐匹克隆 OR 扎来普隆)  AND  (随机 OR 随机对照 OR 随机对照试验)  NOT  (儿童 OR 青少年 OR 婴儿 OR 抑郁 OR 焦虑 OR 癌症 OR 肿瘤 OR 卒中 OR 脑卒中 OR 绝经 OR 围绝经期 OR 妊娠 OR 孕妇 OR 透析 OR 血液透析 OR 疼痛 OR 帕金森 OR 痴呆 OR 精神分裂症 OR 双相情感障碍 OR 睡眠呼吸暂停) | 7 |

**The search strategy (维普)**

| Search number | Query | Results |
| --- | --- | --- |
| 1 | (慢性失眠 OR 慢性失眠障碍 OR 原发性失眠 OR 持续性失眠 OR (失眠 AND 慢性))  AND  (针刺 OR 针灸 OR 毫针 OR 电针 OR 中医药 OR 传统中医药 OR 中药 OR 中药汤剂 OR 汤剂 OR 西药 OR 安眠药 OR 镇静催眠药 OR 苯二氮卓类 OR 非苯二氮卓类 OR 唑吡坦 OR 佐匹克隆 OR 右佐匹克隆 OR 扎来普隆)  AND  (随机 OR 随机对照 OR 对照 OR 临床试验)  NOT  (儿童 OR 青少年 OR 婴儿 OR 抑郁 OR 焦虑 OR 癌症 OR 肿瘤 OR 卒中 OR 脑卒中 OR 绝经 OR 围绝经期 OR 妊娠 OR 孕妇 OR 透析 OR 血液透析 OR 疼痛 OR 帕金森 OR 痴呆 OR 精神分裂症 OR 双相情感障碍 OR 睡眠呼吸暂停) | 35 |

**The search strategy (中国生物医学)**

| Search number | Query | Results |
| --- | --- | --- |
| 1 | ( "(慢性失眠"[常用字段:智能] OR "慢性失眠障碍"[常用字段:智能] OR "原发性失眠"[常用字段:智能] OR "持续性失眠)"[常用字段:智能]) AND (针刺 OR 针灸 OR 电针 OR 毫针 OR 唑吡坦 OR 佐匹克隆 OR 右佐匹克隆 OR 扎来普隆 OR 中药汤剂) AND( "(随机对照试验"[常用字段:智能] OR "随机对照"[常用字段:智能] OR "随机分组)"[常用字段:智能]) NOT( "(儿童"[常用字段:智能] OR "青少年"[常用字段:智能] OR "婴儿"[常用字段:智能] OR "肿瘤"[常用字段:智能] OR "癌症"[常用字段:智能] OR "脑卒中"[常用字段:智能] OR "卒中"[常用字段:智能] OR "孕妇"[常用字段:智能] OR "妊娠"[常用字段:智能] OR "帕金森"[常用字段:智能] OR "痴呆"[常用字段:智能] OR "睡眠呼吸暂停)"[常用字段:智能]) | 640 |
