# Supplementary material for: Efficacy of acupuncture, Chinese herbal medicine, combined acupuncture-herbal therapy, and western medication for adult chronic insomnia: a network meta-analysis
Source: Front Med (Lausanne). 2026 Sep 30;13:1961539. doi: 10.3389/fmed.2026.1961539 (PMC13661500; doi:10.3389/fmed.2026.1961539)
Supplement: Supplementary file 2 [file Supplementaryfile2.docx]

**Table S2** Overview of the studies included in the analysis

| **First author** | **year** | **Region** | **Number of cases** | | **Age** | | **Intervention measures** | | **course** | **Number of lost follow-ups** | | **Outcome indicators** |
| --- | --- | --- | --- | --- | --- | --- | --- | --- | --- | --- | --- | --- |
|  |  |  | **EG** | **CG** | **EG** | **CG** | **EG** | **CG** |  | **EG** | **CG** |  |
| Qiong An | 2015 | China | 46 | 46 | 46.02±1.34 | 46.53±1.49 | Herbal medicine | Western medicine | 15 days | 0 | 0 | Total efficacy |
| Haibo Jia | 2021 | China | 30 | 30 | 43.34±4.72 | 43.01±3.93 | Acupuncture plus herbal medicine | Western medicine | 30 days | 0 | 0 | Total efficacy, PSQI, Adverse reaction |
| Linxiao Zhong | 2019 | China | 44 | 44 | 44.3±3.9 | 45.3±3.2 | Acupuncture | Western medicine | 28 days | 0 | 0 | Total efficacy, PSQI |
| Guohui Lin | 2021 | China | 166 | 166 | 49.7±5.6 | 48.8±4.5 | Acupuncture | Western medicine | 28 days | 0 | 0 | Total efficacy, PSQI |
| Jieling Hu | 2021 | China | 45 | 45 | 51.23±7.04 | 49.23±7.13 | Acupuncture plus herbal medicine | Herbal medicine | 14 days | 0 | 0 | Total efficacy, PSQI |
| Xiaoling Wang | 2005 | China | 32 | 31 | N/A | N/A | Acupuncture | Herbal medicine | 30 days | 0 | 1 | Total efficacy |
| Xinfang He | 2006 | China | 40 | 32 | 39.2±11.7 | 38.9±11.3 | Acupuncture plus herbal medicine | Acupuncture | 24 days | 0 | 8 | Total efficacy |
| Hong Xu | 2003 | China | 45 | 45 | N/A | N/A | Acupuncture plus herbal medicine | Herbal medicine | 35 days | 0 | 0 | Total efficacy |
| Xiaowen Ye | 2006 | China | 42 | 37 | 37±5.4 | 35.1±7.4 | Acupuncture plus herbal medicine | Western medicine | 21 days | 0 | 5 | Total efficacy |
| Lihua Huang | 2023 | China | 40 | 40 | 53.52±2.35 | 54.6±3.83 | Acupuncture plus herbal medicine | Herbal medicine | 28 days | 0 | 0 | Total efficacy, PSQI |
| Weifang Wang | 2013 | China | 40 | 40 | 42.98±2.35 | 43.34±3.2 | Acupuncture | Western medicine | 10 days | 0 | 0 | Total efficacy |
| Zuhuang Zhang | 2015 | China | 62 | 57 | 44.98±11.67 | 43.18±12.08 | Acupuncture | Western medicine | 30 days | 0 | 5 | Total efficacy, PSQI |
| Lei Wang | 2013 | China | 40 | 40 | 45.4±1.62 | 56.0±1.25 | Acupuncture plus herbal medicine | Herbal medicine | 30 days | 0 | 0 | Total efficacy |
| Ting Zhang | 2011 | China | 36 | 36 | 40.3±2.6 | 43.3±3.6 | Acupuncture plus herbal medicine | Herbal medicine | 10 days | 0 | 0 | PSQI |
| Qiang Liu | 2019 | China | 47 | 47 | 51.28±20.31 | 52.31±18.79 | Acupuncture plus herbal medicine | Western medicine | 56 days | 0 | 0 | Total efficacy, Adverse reaction |
| Yan Cui | 2021 | China | 34 | 34 | 54.51±3.36 | 54.39±3.12 | Acupuncture | Western medicine | 10 days | 0 | 0 | Total efficacy, Adverse reaction |
| Yan Pi | 2017 | China | 29 | 29 | 43.34±12.28 | 43.45±13.28 | Acupuncture | Western medicine | 28 days | 1 | 1 | PSQI |
| Jianfang Zhu | 2022 | China | 27 | 26 | 50±3 | 50±2 | Acupuncture | Western medicine | 28 days | 3 | 4 | Total efficacy, PSQI |
| Qiulan Pan | 2005 | China | 56 | 56 | N/A | N/A | Acupuncture | Western medicine | 28 days | 0 | 0 | Total efficacy |
| Zhanxue Ma | 2006 | China | 31 | 31 | 42.5±8.5 | 44.6±9.3 | Acupuncture | Western medicine | 30 days | 0 | 0 | Total efficacy |
| Dingwen Guo | 2020 | China | 20 | 20 | 39.65±10.7 | 42.35±9.59 | Acupuncture plus herbal medicine | Western medicine | 28 days | 0 | 0 | Total efficacy |
| Jianyao Liu | 2014 | China | 50 | 50 | 50.3±2.5 | 55.2±1.5 | Acupuncture plus herbal medicine | Herbal medicine | 30 days | 0 | 0 | Total efficacy |
| Ying Jin | 2016 | China | 24 | 24 | 39.2±16.9 | 38.8±17.7 | Acupuncture plus herbal medicine | Acupuncture | 15 days | 0 | 0 | Total efficacy, PSQI |
| Feng Li | 2014 | China | 30 | 30 | 53.5 | 54.5 | Acupuncture | Western medicine | 14 days | 0 | 0 | Total efficacy, PSQI |
| Xiangdong Ji | 2015 | China | 35 | 35 | 37±11 | 36±13 | Acupuncture | Western medicine | 14 days | 0 | 0 | Total efficacy, PSQI |
| Yuliang Wang | 2021 | China | 30 | 30 | 63.39±4.732 | 64.38±5.342 | Acupuncture plus herbal medicine | Herbal medicine | 21 days | 0 | 0 | Total efficacy, PSQI |
| Xiaolin Yang | 2017 | China | 46 | 46 | 49.91±2.48 | 50.04±2.65 | Acupuncture plus herbal medicine | Western medicine | 28 days | 0 | 0 | Total efficacy, Adverse reaction |
| Hui Xu | 2014 | China | 45 | 45 | 49.11±3.62 | 49.50±4.01 | Acupuncture | Western medicine | 14 days | 0 | 0 | Total efficacy, PSQI, Adverse reaction |
| Zhiwei Tan | 2020 | China | 47 | 47 | 70.03±3.11 | 69.1±6.84 | Acupuncture | Western medicine | 28 days | 0 | 0 | Total efficacy, PSQI |
| Jinzhuo Fan | 2019 | China | 50 | 49 | 68.45±5.30 | 68.52±5.38 | Acupuncture plus herbal medicine | Western medicine | 28 days | 0 | 1 | Total efficacy, Adverse reaction |
| Xingli Huang | 2010 | China | 68 | 66 | N/A | N/A | Acupuncture | Western medicine | 14 days | 0 | 2 | Total efficacy |
| Qiuping Liu | 2025 | China | 25 | 25 | 35.45±3.23 | 35.20±1.05 | Acupuncture plus herbal medicine | Herbal medicine | 14 days | 0 | 0 | Total efficacy, PSQI |
| Siwen Xu | 2015 | China | 30 | 28 | 48.2±13.7 | 45.9±12.5 | Acupuncture plus herbal medicine | Herbal medicine | 20 days | 0 | 2 | Total efficacy |
| Jinxia Ni | 2006 | China | 39 | 37 | N/A | N/A | Acupuncture | Western medicine | 56 days | 1 | 3 | Total efficacy |
| Zhibin Liu | 2007 | China | 80 | 80 | 48.06±6.12 | 49.11±2.24 | Acupuncture | Western medicine | 28 days | 0 | 0 | Total efficacy, PSQI |
| Xinhan Zhu | 2019 | China | 30 | 30 | 42.08±13.11 | 44.51±11.47 | Acupuncture | Western medicine | 28 days | 0 | 0 | Total efficacy, PSQI |
| Peifang Li | 2013 | China | 50 | 50 | 44.26±6.12 | 45.35±5.78 | Acupuncture | Western medicine | 28 days | 0 | 0 | Total efficacy, PSQI |
| Siwei Zhang | 2013 | China | 30 | 30 | 45.43±2.66 | 49.23±2.50 | Acupuncture plus herbal medicine | Herbal medicine | 14 days | 0 | 0 | Total efficacy |
| Lijun Ba | 2023 | China | 42 | 42 | 62.42±6.25 | 62.38±6.21 | Acupuncture plus herbal medicine | Western medicine | 28 days | 0 | 0 | Total efficacy |
| Xuefen Liao | 2013 | China | 30 | 30 | 47.4±9.21 | 47.7±8.92 | Acupuncture plus herbal medicine | Herbal medicine | 35 days | 0 | 0 | Total efficacy |
| Xiaowei Ma | 2013 | China | 50 | 50 | 41.2±4.5 | 40.6±4.8 | Acupuncture | Western medicine | 28 days | 0 | 0 | Total efficacy |
| Wen Yao | 2014 | China | 30 | 30 | 49.8±13.2 | 51.2±12.5 | Acupuncture | Western medicine | 10 days | 0 | 0 | Total efficacy, PSQI |
| Jinhong Hao | 2011 | China | 49 | 48 | 46.6±5.6 | 48.6±4.7 | Herbal medicine | Western medicine | 14 days | 0 | 1 | Total efficacy |
| Lili Hu | 2024 | China | 75 | 75 | 52.21±5.05 | 52.17±5.24 | Acupuncture | Western medicine | 60 days | 0 | 0 | Total efficacy, PSQI |
| Chujiao Tian | 2024 | China | 52 | 52 | N/A | N/A | Herbal medicine | Western medicine | 28 days | 0 | 0 | Total efficacy |
| Guilian Chen | 2020 | China | 38 | 38 | 41.12±8.45 | 40.87±8.36 | Acupuncture | Herbal medicine | 21 days | 0 | 0 | Total efficacy, PSQI |
| Haiyan Wu | 2014 | China | 48 | 48 | 52.1±8.9 | 51.8±7.4 | Acupuncture plus herbal medicine | Western medicine | 28 days | 0 | 0 | Total efficacy, PSQI |
| Dan Xu | 2020 | China | 40 | 43 | 45.05±10.88 | 47.40±9.49 | Herbal medicine | Western medicine | 14 days | 3 | 0 | Total efficacy, PSQI |
| Xiaojun Zhu | 2025 | China | 57 | 57 | 57.96±9.01 | 56.77±9.36 | Herbal medicine | Western medicine | 28 days | 0 | 0 | Total efficacy, PSQI |
| Lanhua Yin | 2013 | China | 30 | 30 | 45.2±4.68 | 45.6±4.88 | Herbal medicine | Western medicine | 7 days | 0 | 0 | Total efficacy, PSQI |
| Yongyi Xu | 2020 | China | 30 | 30 | 48.3±7.8 | 47.4±8.1 | Acupuncture | Western medicine | 28 days | 0 | 0 | Total efficacy, PSQI |
| Xiaojun Jiang | 2022 | China | 42 | 42 | 48.17±6.64 | 47.63±7.02 | Acupuncture | Western medicine | 28 days | 0 | 0 | PSQI, Adverse reaction |
| Pei Li | 2017 | China | 32 | 31 | 41.5±6.1 | 42.2±6.5 | Acupuncture plus herbal medicine | Western medicine | 28 days | 0 | 1 | Total efficacy, Adverse reaction |
| Qihui Zhang | 2006 | China | 36 | 32 | 50.11±3.32 | 49.75±3.18 | Herbal medicine | Western medicine | 28 days | 4 | 8 | Total efficacy, Adverse reaction |
| Yanjun Wang | 2016 | China | 34 | 34 | 53±13.43 | 53±11.37 | Acupuncture | Western medicine | 28 days | 0 | 0 | Total efficacy, PSQI |
| Xiuling Chen | 2011 | China | 38 | 32 | 48±6 | 48±7 | Acupuncture | Western medicine | 27 days | 0 | 6 | Total efficacy, Adverse reaction |
| Kai Wang | 2006 | China | 40 | 40 | N/A | N/A | Acupuncture plus herbal medicine | Western medicine | 7 days | 0 | 0 | Total efficacy |
| Yong Zou | 2008 | China | 60 | 58 | N/A | N/A | Acupuncture | Western medicine | 25 days | 0 | 2 | Total efficacy |
| Duo Li | 2024 | China | 40 | 40 | 47.41±4.30 | 53.70±1.60 | Acupuncture plus herbal medicine | Western medicine | 28 days | 0 | 0 | Total efficacy, PSQI |

Appendix 2. List of the included studies (non-English publications)

**The following documents(17-75) are all non-English publications and all come from China.**

17.	An Q, Zhong L. Zuogui pill for refractory insomnia: a randomized parallel controlled study. J Pract Tradit Chin Intern Med. 2015;29(2):74-6.

18.	Ba L. Effect of acupuncture combined with Guipi Decoction on sleep quality in patients with insomnia of heart-spleen deficiency. Guide China Med. 2023;21(9):118-20.

19.	Chen G, Pan H. Clinical observation on Guipi Decoction combined with acupuncture for 38 cases of insomnia of heart-spleen deficiency type. Chin J Ethnomed Ethnopharm. 2020;29(15):97-9.

20.	Chen X, Xu K, Qin X. Observation on the therapeutic effect of electroacupuncture for female perimenopausal insomnia. Shanghai J Acupunct Moxibustion. 2011;30(6):366-7.

21.	Cui Y. Effect of acupuncture plus auricular point sticking combined with Chinese herbal medicine on intractable insomnia. Chin J Urban Rural Enterp Health. 2021;36(10):126-7.

22.	Fan J, Xiong J, He X. Clinical study of Yangxin Hewei Yimian Decoction combined with acupuncture at Anmian point for senile insomnia. Chin Arch Tradit Chin Med. 2019;37(2):466-9.

23.	Guo D, Wu Q. Acupuncture combined with modified Xiaochaihu Decoction for 20 cases of liver-qi stagnation insomnia. Res Tradit Chin Med. 2020;33(5):50-2.

24.	Hao J. Clinical observation on Jieyu Anshen Decoction for insomnia in menopausal women. Chin Arch Tradit Chin Med. 2011;29(7):1691-3.

25.	He X, Xu H. Observation on the therapeutic effect of acupuncture combined with medicine in the treatment of insomnia. J Chin Med Mater. 2006;29(2):203-4.

26.	Hu J, Tian H. Effect of spirit-regulating acupuncture on sleep quality, TCM syndrome scores and SDS scores in patients with insomnia of yin deficiency with effulgent fire type. J Sichuan Tradit Chin Med. 2021;39(11):211-4.

27.	Hu L, Wang J. Clinical effect of electroacupuncture at Jiaji points for insomnia with depressive disorder analyzed by resting-state functional MRI. Pract Clin J Integr Tradit Chin West Med. 2024;24(1):56-9.

28.	Huang L, Zhang Y. Clinical study on acupuncture combined with medicine in treating menopausal insomnia of yin deficiency and fire hyperactivity type. Guangming J Chin Med. 2023;38(16):3088-91.

29.	Huang X, Wang P. Clinical observation of eye acupuncture for intractable insomnia. Liaoning J Tradit Chin Med. 2010;37(9):1801-2.

30.	Ji X, Wang Q, Zhu W. Effect of acupuncture on the expression of different neurotransmitters in patients with liver-depression-fire type insomnia. Chin Acupunct Moxibustion. 2015;35(6):549-52.

31.	Jia H, Wang C, Xin H, Li J, Liang X, Duo H. Clinical efficacy evaluation of self-made Anshen Yangxue Decoction combined with warm needling and auricular acupoint therapy for refractory insomnia. J Hebei Tradit Chin Med Pharmacol. 2021;36(1):43-6.

32.	Jiang X, Chen J, Zhu S. A randomized controlled trial on the efficacy and safety of mind-body regulating acupuncture for chronic insomnia. Smart Healthc. 2022;8(33):178-81.

33.	Jin Y, Hai Y. Acupuncture combined with Guipi Decoction for heart-spleen deficiency type insomnia: a randomized parallel controlled study. J Pract Tradit Chin Intern Med. 2016;30(6):84-7.

34.	Li D, Zhang G, Yue Y. Clinical efficacy observation on Baihe Dihuang Decoction combined with acupuncture for insomnia of yin deficiency constitution. Inner Mongolia J Tradit Chin Med. 2024;43(2):11-3.

35.	Li F, Ren Y, An S, Wang D. Clinical observation of acupuncture at Jiaji (EX-B2) points for insomnia. J Clin Acupunct Moxib. 2014;30(3):16-8.

36.	Li P, Ding N, Xiang S. Clinical observation of yin-yang harmonization therapy for insomnia of heart-kidney disharmony type. Shanxi J Tradit Chin Med. 2017;33(8):12-3.

37.	Li P, Liu X. Clinical observation on Tongdu Tiaoshen acupuncture for insomnia. J Clin Acupunct Moxibustion. 2013;29(4):17-8.

38.	Liao X. Randomized parallel controlled study of Bo Zhiyun's abdominal acupuncture combined with Qiju Dihuang Decoction for insomnia of liver-kidney yin deficiency in middle-aged and elderly patients. J Pract Tradit Chin Intern Med. 2013;27(2):120-1.

39.	Lin G. Clinical effect of traditional Chinese medicine combined with acupuncture and auricular point pressing with beans in the treatment of insomnia. Nei Mongol J Tradit Chin Med. 2021;40(1):115-6.

40.	Liu J. Acupuncture combined with Ningshen Decoction No. 2 for liver-fire disturbing the heart type insomnia: a randomized parallel controlled study. J Pract Tradit Chin Intern Med. 2014;28(11):129-31.

41.	Liu Q. Clinical study on acupuncture combined with Yangxin Anshen Decoction in treating insomnia of heart-spleen deficiency type. Inner Mongol J Tradit Chin Med. 2019;38(6):101-2.

42.	Liu Q, Mao R, Hu Q, Huang Y. Modified Xiaoyao Powder combined with acupuncture for menstrual insomnia of liver depression and spleen deficiency type. Nei Mongol J Tradit Chin Med. 2025;44(2):72-3,99.

43.	Liu Z, Niu W. Scalp hairline zone row needling for 160 cases of insomnia. Shaanxi J Tradit Chin Med. 2007;28(6):716-7.

44.	Ma X, Deng L, Kuang J, Ou W, Cao Y. Observation on the effect of acupuncture on the Ren meridian for insomnia. Guide China Med. 2013;11(36):198-9.

45.	Ma Z, Li G, Duan H. Observation on the efficacy of acupuncture for insomnia in 31 cases. Guangming J Chin Med. 2006;21(4):25-6.

46.	Ni J, Zhu W. Clinical comparative study of scalp point penetration needling as main therapy for 76 cases of insomnia. J Clin Acupunct Moxibustion. 2006;22(12):35-6.

47.	Pan Q, Zhang L, Yang J, He Y. Analysis of the efficacy of acupuncture for intractable insomnia. Chin Trop Med. 2005;5(8):1705.

48.	Pi Y, Tang Y, Wang Z, Wang L. Effect of acupuncture treatment for primary insomnia on each factor of the Pittsburgh Sleep Quality Index. J Sichuan Tradit Chin Med. 2017;35(11):188-91.

49.	Tan Z. Clinical observation on yin-zhong-yin-yang acupuncture method for senile insomnia. Chin Manip Rehabil Med. 2020;11(19):25-6.

50.	Tian C, Wang L, Wang S, Zou T, Liu Z, Li S. A randomized controlled trial of Hewei Anshen Decoction for 52 male patients with stress insomnia. J Tradit Chin Med. 2024;65(22):2326-31.

51.	Wang K. Clinical observation on electroacupuncture combined with Chinese herbal medicine for insomnia. J Clin Acupunct Moxibustion. 2006;22(5):34-5.

52.	Wang L. Randomized parallel controlled study on direct acupuncture combined with Ningshen Decoction in treating insomnia of liver fire disturbing heart type. J Pract Tradit Chin Intern Med. 2013;27(4):114-5.

53.	Wang W. Clinical observation on the therapeutic effect of acupuncture in treating insomnia. Mod Diagn Treat. 2013;24(6):1285-6.

54.	Wang X. Observation on the therapeutic effect of sedation and tranquilization acupuncture in 63 patients with insomnia. Shanghai J Acupunct Moxibustion. 2005;24(6):21-2.

55.	Wang Y, Zhang L, Han Y, Li P. Observation on the therapeutic effect of Du-regulating and mind-tranquilizing acupuncture for insomnia. J Acupunct Tuina Sci. 2016(4):274-8.

56.	Wang Y, Zhang R, Wang C, Hu H, Xiao M, Sui R. Acupuncture at Dong's extraordinary points combined with Sini Powder for liver-depression and spleen-deficiency type insomnia in the elderly: a clinical observation. Chin J Geriatr Care. 2021;19(3):17-9.

57.	Wu H. Modified Guipi Decoction combined with body acupuncture for 48 cases of insomnia of heart-spleen deficiency type. J Tradit Chin Med Res. 2014;27(4):51-2.

58.	Xu D, Gao H. Clinical observation of Ganmai Dazao Decoction combined with Suanzaoren Decoction for insomnia of yin deficiency with fire hyperactivity type. Guangming J Chin Med. 2020;35(21):3376-8.

59.	Xu H, Hu Z, Wang Q. Clinical study on acupuncture combined with medicine in the treatment of insomnia. Liaoning J Tradit Chin Med. 2003;30(7):570-1.

60.	Xu H, Wang P, He W. Clinical observation of eye acupuncture therapy for insomnia. J Liaoning Univ Tradit Chin Med. 2014;16(8):109-11.

61.	Xu S. Wendan Anmian Decoction combined with acupuncture for insomnia of phlegm-heat disturbing the heart type: a randomized parallel controlled study. J Pract Tradit Chin Intern Med. 2015;29(5):156-7.

62.	Xu Y, Lin X. Clinical study of acupuncture at six spirit-regulating points combined with three sleep points for primary insomnia of liver-fire disturbing heart type. J New Chin Med. 2020;52(3):151-3.

63.	Yang X. Efficacy observation of Yueju Pill combined with superficial acupuncture for perimenopausal insomnia. Guangming J Chin Med. 2017;32(1):84-5.

64.	Yao W, Li D, Wu Q, Yan J, Ye H. Clinical observation on mind-regulating acupuncture with extraordinary points for insomnia. Clin J Tradit Chin Med. 2014;6(04):4-6.

65.	Ye X, Dai W. Observation on the therapeutic effect of acupuncture combined with medicine in the treatment of insomnia. J Yunnan Coll Tradit Chin Med. 2006;29(4):26-7.

66.	Yin L. Eryin Decoction for diabetic insomnia: a randomized parallel controlled study. J Pract Tradit Chin Intern Med. 2013;27(6):16-8.

67.	Zhang Q. Clinical observation on liver-regulating and spleen-harmonizing Chinese herbal medicine for 36 cases of perimenopausal insomnia. J Tradit Chin Med. 2006;47(9):678-80.

68.	Zhang S, Tian W. Randomized parallel controlled study of Tian Weizhu's eye acupuncture combined with Wendan Decoction for insomnia. J Pract Tradit Chin Intern Med. 2013;27(12):24-5.

69.	Zhang T. Acupuncture and moxibustion combined with Chinese herbal medicine in treating 36 cases of insomnia of heart-spleen deficiency type. J Extern Ther Tradit Chin Med. 2011;20(1):26-7.

70.	Zhang Z, Liu Z. Clinical observation on the therapeutic effect of acupuncture on insomnia patients and its influence on the levels of inflammatory factors. Shaanxi J Tradit Chin Med. 2015;36(10):1420-2.

71.	Zhong L. Observation on the therapeutic effect of traditional Chinese medicine acupuncture in patients with insomnia. World J Sleep Med. 2019;6(8):1066-7.

72.	Zhu J, Liu T, Hu F, Sheng J, Jin Y. Acupuncture for perimenopausal early-awakening insomnia: a randomized controlled trial. Chin Acupunct Moxibustion. 2022;42(6):608-12.

73.	Zhu X, Zeng H, Zhu S, Zhu S. Clinical observation of Fuzi Decoction combined with Guizhi Gancao Longgu Muli Decoction for insomnia of heart-kidney yang deficiency type. Chin Arch Tradit Chin Med. 2025;43(11):200-3.

74.	Zhu X, Zhang F, Dai J, Dou Z, Yang M, Zhu R, et al. Clinical study on Tongdu Tiaoshen acupuncture for insomnia. Lishizhen Med Mater Med Res. 2019;30(2):389-90.

75.	Zou Y. Clinical observation on mind-tranquilizing and will-stabilizing electroacupuncture for psychogenic insomnia. Heilongjiang J Tradit Chin Med. 2008;37(1):40-1.
