# Supplementary material for: Efficacy of acupuncture, Chinese herbal medicine, combined acupuncture-herbal therapy, and western medication for adult chronic insomnia: a network meta-analysis
Source: Front Med (Lausanne). 2026 Sep 30;13:1961539. doi: 10.3389/fmed.2026.1961539 (PMC13661500; doi:10.3389/fmed.2026.1961539)
Supplement: Supplementary file 3 [file Supplementaryfile3.docx]

****Appendix 3. SUCRA ranking results****

**Total efficacy (TE)**

The SUCRA ranking of TE was as follows: acupuncture plus herbal medicine (92.0%) > acupuncture (74.7%) > herbal medicine (33.1%) > Western medicine (0.2%).

**Pittsburgh Sleep Quality Index (PSQI)**

The SUCRA ranking for PSQI was as follows: acupuncture (82.0%) > acupuncture plus herbal medicine (76.8%) > herbal medicine (36.4%) > Western medicine (4.9%).

**Adverse events (AEs)**

The SUCRA ranking for AEs, where higher values indicate a lower risk of adverse events (better safety profile), was as follows: acupuncture (78.8%) > herbal medicine (76.0%) > acupuncture plus herbal medicine (45.2%) > Western medicine (<0.1%).
