# Supplementary material for: Efficacy of acupuncture, Chinese herbal medicine, combined acupuncture-herbal therapy, and western medication for adult chronic insomnia: a network meta-analysis
Source: Front Med (Lausanne). 2026 Sep 30;13:1961539. doi: 10.3389/fmed.2026.1961539 (PMC13661500; doi:10.3389/fmed.2026.1961539)
Supplement: Supplementary file 4 [file Supplementaryfile4.docx]

# Appendix 4 Full results of the node-splitting analysis

Node-splitting was performed using the mtc.nodesplit function in the GeMTC package (random-effects model; 5,000 adaptation iterations, 100,000 sampling iterations, thinning = 1, 4 chains). For each comparison informed by both direct and indirect evidence, the tables report the direct estimate, indirect estimate, network (combined) estimate, and the inconsistency P value; P > 0.05 indicates no statistically significant inconsistency between direct and indirect evidence.

## Total efficacy (TE; log RR scale)

| **Comparison** | **Inconsistency P value** | **Direct estimate (95% CrI)** | **Indirect estimate (95% CrI)** | **Network estimate (95% CrI)** |
| --- | --- | --- | --- | --- |
| Acupuncture vs Western medicine | 0.607 | 0.18 (0.13, 0.23) | 0.14 (-0.018, 0.29) | 0.17 (0.13, 0.22) |
| Herbal medicine vs Western medicine | 0.022 | 0.13 (0.039, 0.22) | -0.025 (-0.12, 0.070) | 0.056 (-0.011, 0.12) |
| Acupuncture plus herbal medicine vs Western medicine | 0.008 | 0.14 (0.069, 0.22) | 0.31 (0.21, 0.42) | 0.20 (0.14, 0.27) |
| Herbal medicine vs Acupuncture | 0.544 | -0.17 (-0.38, 0.020) | -0.11 (-0.19, -0.022) | -0.12 (-0.20, -0.041) |
| Acupuncture plus herbal medicine vs Acupuncture | 0.168 | 0.17 (-0.043, 0.40) | 0.0095 (-0.070, 0.090) | 0.029 (-0.045, 0.11) |
| Acupuncture plus herbal medicine vs Herbal medicine | 0.050 | 0.19 (0.11, 0.28) | 0.059 (-0.051, 0.17) | 0.15 (0.083, 0.22) |

For TE, statistically significant inconsistency between direct and indirect evidence was detected for two comparisons (herbal medicine vs Western medicine, P = 0.022; acupuncture plus herbal medicine vs Western medicine, P = 0.008), and a borderline result was observed for acupuncture plus herbal medicine vs herbal medicine (P = 0.050); the remaining comparisons showed no significant inconsistency.

## Pittsburgh Sleep Quality Index (PSQI; MD scale)

| **Comparison** | **Inconsistency P value** | **Direct estimate (95% CrI)** | **Indirect estimate (95% CrI)** | **Network estimate (95% CrI)** |
| --- | --- | --- | --- | --- |
| Acupuncture vs Western medicine | 0.588 | -2.4 (-3.6, -1.1) | -3.5 (-7.6, 0.59) | -2.5 (-3.6, -1.3) |
| Herbal medicine vs Western medicine | 0.968 | -1.1 (-4.0, 1.9) | -1.2 (-4.0, 1.7) | -1.1 (-3.2, 0.91) |
| Acupuncture plus herbal medicine vs Western medicine | 0.575 | -2.8 (-5.8, 0.077) | -1.7 (-4.6, 1.2) | -2.3 (-4.3, -0.25) |
| Herbal medicine vs Acupuncture | 0.252 | 3.9 (-1.1, 8.9) | 0.73 (-1.7, 3.2) | 1.3 (-0.85, 3.5) |
| Acupuncture plus herbal medicine vs Acupuncture | 0.685 | -0.69 (-5.7, 4.3) | 0.41 (-2.1, 2.9) | 0.19 (-2.0, 2.4) |
| Acupuncture plus herbal medicine vs Herbal medicine | 0.369 | -0.61 (-2.8, 1.6) | -2.5 (-6.0, 1.1) | -1.1 (-3.0, 0.74) |

For PSQI, all node-splitting P values exceeded 0.05 (range 0.252-0.968), indicating no statistically significant inconsistency between direct and indirect evidence for any comparison.

## Adverse events (AEs)

Node-splitting was not applicable to the AE outcome: because only 10 trials reported adverse events, no comparison in the AE network was informed by both direct and indirect evidence (i.e., the network contained no closed loops), and inconsistency could therefore not be formally assessed for this outcome.
